# Identification of *Plasmodium falciparum* DNA Repair Protein Mre11 with an Evolutionarily Conserved Nuclease Function

**DOI:** 10.1371/journal.pone.0125358

**Published:** 2015-05-04

**Authors:** Sugith Babu Badugu, Shaik Abdul Nabi, Pratap Vaidyam, Shyamasree Laskar, Sunanda Bhattacharyya, Mrinal Kanti Bhattacharyya

**Affiliations:** 1 Department of Biochemistry, School of Life Sciences, University of Hyderabad, Hyderabad, India; 2 Department of Biotechnology and Bioinformatics, School of Life Sciences, University of Hyderabad, Hyderabad, India; Tulane University Health Sciences Center, UNITED STATES

## Abstract

The eukaryotic Meiotic Recombination protein 11 (Mre11) plays pivotal roles in the DNA damage response (DDR). Specifically, Mre11 senses and signals DNA double strand breaks (DSB) and facilitates their repair through effector proteins belonging to either homologous recombination (HR) or non-homologous end joining (NHEJ) repair mechanisms. In the human malaria parasite *Plasmodium falciparum*, HR and alternative-NHEJ have been identified; however, little is known about the upstream factors involved in the DDR of this organism. In this report, we identify a putative ortholog of Mre11 in *P*. *falciparum* (PfalMre11) that shares 22% sequence similarity to human Mre11. Homology modeling reveals striking structural resemblance of the predicted PfalMre11 nuclease domain to the nuclease domain of *Saccharomyces cerevisiae* Mre11 (ScMre11). Complementation analyses reveal functional conservation of PfalMre11 nuclease activity as demonstrated by the ability of the PfalMre11 nuclease domain, in conjunction with the C-terminal domain of ScMre11, to functionally complement an *mre11* deficient yeast strain. Functional complementation was virtually abrogated by an amino acid substitution in the PfalMre11 nuclease domain (D398N). PfalMre11 is abundant in the mitotically active trophozoite and schizont stages of *P*. *falciparum* and is up-regulated in response to DNA damage, suggesting a role in the DDR. PfalMre11 exhibits physical interaction with PfalRad50. In addition, yeast 2-hybrid studies show that PfalMre11 interacts with ScRad50 and ScXrs2, two important components of the well characterized Mre11-Rad50-Xrs2 complex which is involved in DDR signaling and repair in *S*. *cerevisiae*, further supporting a role for PfalMre11 in the DDR. Taken together, these findings provide evidence that PfalMre11 is an evolutionarily conserved component of the DDR in *Plasmodium*.

## Introduction

Malaria continues to be one of the deadliest infectious diseases worldwide, resulting in nearly several millions deaths annually. *P*. *falciparum*, a mosquito-borne protozoan parasite, is responsible for most malaria deaths. Infection with *P*. *falciparum* can lead to serious medical complications, including cerebral malaria, as well as increased risk for long-term neurological and cognitive impairments. Currently, no malaria vaccine is available but effective treatments do exist. However, the rapid emergence of drug-resistant *P*. *falciparum* [1] underscores the urgent need for additional pharmacotherapies that are effective.

DNA repair pathways represent potential sources of new targets for treatment of *Plasmodium* infections, given that even a single un-repaired DSB leads to death of a unicellular organism [2]. In fact, previous research has shown that the parasite is susceptible to extensive DSBs caused by exposure to radiomimetic drugs, accumulation of free heme, innate host immune responses and DNA replication errors [3–5]. In eukaryotes, DSBs activate the DDR pathway which recognizes and processes DSBs, activates cell signaling pathways, and facilitates repair by either NHEJ or HR. In *P*. *falciparum*, HR has been identified and characterized [6, 7], is the predominant DSB repair mechanism [8], and is essential for repairing DSBs [9]. Interestingly, *P*. *falciparum* appears to lack the canonical NHEJ pathway consisting of the Ku heterodimer, DNA-PKc, DNA ligase VI and XRCC4, and alternative NHEJ (A-NHEJ) is utilized at a very low frequency [8]. The presence of HR and A-NHEJ in *Plasmodium* suggest potential overlap with DNA repair pathways in well-characterized eukaryotes; however, the factors involved in *Plasmodium* DDR remain largely unknown and orthologs of key eukaryotic DDR factors including ATM (yeast Mec1), ATR (yeast Tel1), Chk1, Chk2 (yeast Rad53) and Mre11 are yet to be identified and characterized.

The multi-functional Mre11-Rad50-Xrs2/Nbs1 (MRX/N) complex (*NBS1* is the vertebrate ortholog of yeast *XRS2*) plays pivotal roles in DNA repair [10] through NHEJ and mitotic HR. MRX is also required for the formation and processing of DSBs necessary for the proper disjunction of paired chromatids during meiotic homologous recombination [11, 12]. During mitotic DSB repair, the MRX complex has two distinct roles: (a) it acts in DNA damage signaling as an upstream DNA damage sensor and modifier in the ATM (Tel1 in yeast) pathway [12], where it activates Tel1p, which in turn specifically phosphorylates Mre11p and Xrs2p in response to DNA damage; and (b) it activates DNA repair, possibly through Mre11p nucleolytic processing [13]. Mre11p has multiple *in vitro* nuclease activities including 3’-5’ exonuclease on dsDNA substrate and endonuclease activity on ssDNA. However, none of these activities explain the generation of MRX dependent 3’ overhangs found *in vivo* during mitotic and meiotic DSB processing [14–16]. It has been speculated that the limited DNA unwinding activity of Mre11-Rad50-Nbs complex in conjunction with the multiple nuclease activities of Mre11p may be responsible for 3’ overhang generation [14]. A recent study showed that the repair choice between NHEJ and HR is directed by distinct Mre11 nuclease activities. While, inhibition of endonuclease activity promoted NHEJ in lieu of HR, inhibition of exonuclease activities conferred a general repair defect [17]. Mre11p nuclease activities are confined to its N-terminal domain, while the C-terminal region contains an Xrs2p binding site and confers Tel1p-mediated DDR activity that is separable from the essential nuclease activity in trans [18]. In addition to its role in DSB repair, Mre11 plays important roles in several aspects of telomere maintenance [19–30]. For example, Mre11p nuclease activity and DNA damage signaling are both required for telomerase mediated telomere formation [18].

Among the protozoan parasites, Mre11 has been identified and characterized in *Trypanosoma brucei* and shown to influence HR and DSB repair [31]. However, it is dispensable for HR mediated VSG gene duplication [32]. In this study, we report molecular cloning of *Plasmodium falciparum* Mre11 *(PfalMRE11)* and show up-regulation of PfalMre11 in response to DNA damage. Additionally, through yeast complementation experiments, we demonstrate that PfalMre11 possesses nuclease activity at its amino-terminal domain. Finally, we show that PfalMre11 interacts ScXrs2, suggesting involvement of PfalMre11 in *Plasmodium* DDR.

## Materials and Methods

### Parasite culture and Methyl Methanesulfonate (MMS) treatment

*P*. *falciparum 3D7* culture was maintained in RPMI1640 media (5% hematocrit) supplemented with 1% Albumax (Invitrogen) and 0.005% hypoxanthine at 37°C using the candle jar method. Parasite cultures were divided into two equal portions: one portion was treated with 0.005% MMS for six hours and a non-MMS treated portion was grown in parallel for the same amount of time. Total RNA/ protein were isolated from both treated and untreated cultures for RT-PCR and Western analysis.

### Plasmids

Sequences of all PCR primers used in this paper are presented in S1 Table. Using *P*. *falciparum* genomic DNA as a template, full length *PfalMRE11* was amplified using OMKB23 and OMKB24 as the forward primer and the reverse primer, respectively, both of which had BamHI flanking sequences. The PCR amplified product was cloned in 2μ yeast expression vector pTA [33]. Similarly, full length *ScMRE11* and the *ScMRE11* C-terminal domain (378 amino acids) were amplified using *S*. *cerevisiae* genomic DNA as a template and the primer-pairs OMKB84-OMKB85 and OMKB161-OMKB85, respectively. Each primer-pair contained a BamHI flanking sequence in the forward primer and PstI flanking sequence in the reverse primer. The amplified products were cloned into the pTA vector. The cloned vectors with full length and C-terminal domain of *ScMRE11* are referred as *ScMRE11* and *ScMRE11C*, respectively. Next, we constructed a chimera with the N-terminal domain of *PfalMRE11* (832 amino acids) fused to the C-terminal domain (378 amino acids) of *ScMre11*. This was done by amplifying a 2,496 bp stretch of DNA corresponding to the N-terminal region of PfalMre11 with primer-pair OMKB23-OMKB100 (HindIII site), and amplifying a 1,134 bp stretch of DNA corresponding to the C-terminal region of ScMre11 with the primer-pair OMKB98 (HindIII site)-OMKB85 (PstI site). The PCR amplified products were fused and cloned into pTA vector and the resulting plasmid is referred in this paper as as *Chimera 1*. To create an N-terminal deletion of 343 AA in *Chimera* 1, i.e. Chimera *1 (∆N343)*, we used *Chimera 1* as template and primer-pair OMKB99 (BamHI site)-OMKB85 (PstI site) to amplify a 2,601 bp fragment corresponding to the *PfalMRE11* nuclease domain only. Next, we created a point mutation (*D398N*) in the nuclease domain of *PfalMRE11* using a splice overlap extension technique with *Chimera* 1 as template and the overlapping primers OMKB299 and OMKB298, to generate the construct *Chimera 1 (D398N)*. We also constructed another chimera in which the nuclease domain of ScMre11 (256 amino acids) was fused to the C-terminal domain of PfalMre11 (406 amino acids), creating *Chimera 2*. This was performed by amplifying 768 bp of N-terminal *SCMRE11* with the primer-pair OMKB84 (BamHI)-OMKB154 (HindIII), amplifying 1,218 base pairs of C-terminal *PfalMRE11* with the primer-pair OMKB153 (HindIII), OMKB89 (PstI), fusing the PCR two products and cloning the resulting fragment into a pTA vector. *Scmre11(D56N)* was PCR amplified from genomic DNA isolated from the yeast strain MKB4 using primer-pair OMKB84-OMKB85 and the resulting fragment was cloned into the pTA vector.

To generate a Mre11 plasmid for antibody production, we expressed the C-terminal domain of PfalMre11 in bacteria. This was done by amplifying a 1,131 bp fragment representing the C-terminus of *PfalMRE11* using the primer pair OMKB188 (BamHI)-OMKB166 (SalI), and cloning the product into the pET28a vector (Novagen). All the recombinant plasmids were confirmed by DNA sequencing.

To create pRS413-kanMX6 plasmid for end joining assays, the kanMX6 cassette was excised from pFA6a-kanMX6 by Not I digestion and cloned into the Not I site of the plasmid pRS413.

For yeast two hybrid analysis, full length *PfalMRE11* was subcloned into the bait vector pGBDU-C1 and prey vector pGAD-C1 [34] to generate *PfalMRE11*-BD and *PfalMRE11*-AD, respectively. To study the interaction between PfalMre11 with ScXrs2 or ScRad50, *ScXRS2* and *ScRAD50* were cloned individually into the bait vector. *ScXRS2* was amplified using *S*. *cerevisiae* genomic DNA and the forward-reverse primer pair OMKB262-OMKB263, both of which had BamHI flanking sequences, and the PCR product cloned into a bait vector with an N-terminal *GAL4* DNA binding domain, generating an *ScXRS2*-BD fusion. Similarly, an *ScRAD50*-BD fusion was created by amplification of *ScRAD50* from *S*. *cerevisiae* genomic DNA with the primer pair OMKB164 (BamHI), OMKB165 (EcoRI) and cloning of the PCR fragment into the bait vector. *Chimera 1* was subcloned into a prey vector with an N-terminal *GAL4* activation domain to generate the *Chimera1*-AD fusion. The yeast *KU80* ORF was amplified from *S*. *cerevisiae* genomic DNA using OMKB210 (BamHI)-OMKB75 (SalI) primers and cloned into the pGBDUC1 vector. The *ScMRE11* gene was excised from the pTA vector and sub-cloned into the BamHI and PstI sites of the bait and the prey vectors. The *PfRAD50* ORF was PCR amplified from *P*. *falciparum* 3D7 genomic DNA using OMKB167 (EcoRI) and OMKB326 (PstI) primers and cloned into pGBDUC1 vector.

### Yeast strains

Genotypes of the yeast strains used in this study are given in S2 Table.

The yeast expression vectors harboring *PfalMRE11*, *ScMRE11*, *ScMRE11C*, *Chimera 1*, *Chimera 1 (∆N343)*, *Chimera 1 (D398N)* and *Chimera 2* were transformed into the *∆mre11* strain, MKB7, to generate the strains BSB3, BSB2, BSB6, BSB4, BSB7, PVY1 and BSB5, respectively. The empty pTA vector was transformed in W303α and MKB7 to generate BSB1 and BSB8, respectively. Strain PJ69-4A was used to study yeast two hybrid interactions. Initially, all bait fusion constructs were transformed into PJ69-4A and the transformants were selected on media lacking uracil to generate the strains BSB15, BSB22 and SAN1. As a control, the empty bait vector pGBDU-C1 was transformed into PJ69-4A to generate BSB14. Next, we transformed the empty prey vector into each of the strains BSB14, BSB15, BSB22 and SAN1 to generate BSB18, BSB20, BSB23 and SAN2, respectively. Similarly a prey-*PfalMRE11* fusion construct was transformed into each of the strains BSB14, BSB15, BSB22 and SAN1, generating BSB19, BSB21, BSB25 and SAN3, respectively. Prey-*ScMRE11* fusion construct was transformed into BSB22 and SAN1 to generate BSB24 and SAN4, respectively. Finally, the prey-*Chimera 1* fusion construct was transformed into BSB22 and SAN1 to generate BSB26 and SAN5, respectively. Strains SAN7-SAN10, BSB28, BSB30, BSB32 and BSB33 were also created in the similar fashion. Transformants carrying both the bait and prey fusion constructs were selected using media lacking uracil and leucine.

### Yeast two hybrid analysis

Bait plasmids that were transformed into PJ69-4A were checked for self activation by plating on media lacking uracil and adenine; lack of growth ensured that the bait fusions did not lead to self activation. We performed yeast two hybrid analysis according to the published protocol [9]. Briefly, we analyzed the interactions between bait and prey fusion constructs by checking the growth of each of the strains BSB18-BSB21, BSB23-26, SAN2, SAN3 and SAN5 on media lacking uracil, leucine and adenine as well as on media lacking uracil, leucine and histidine. Growth on plates containing these media was scored after incubation for 5 days at 30°C.

### PfalMre11 protein expression and antibody generation

An N terminal His_6_ tag pET28a:*PfalMRE11C* construct was transformed into *Escherichia coli* strain Rosetta (DE3). Expression of the recombinant protein was performed as described [35]. Briefly, cells were grown in LB media containing chloramphenicol and kanamycin at 37°C until OD_600_ 0.8. Recombinant protein expression was induced by addition of 1 mM IPTG and incubated for 4 h at 37°C. PfalMre11C protein expression was visualized by running the bacterial lysates on a 10% SDS-PAGE gel. The protein band corresponding to PfalMre11 was excised from the gel and used for immunizing rabbits for antibody generation.

### Antibodies and Western blot analysis

The anti-His antibody (Santa cruz Biotechnology Inc., CA) and HRP-conjugated anti-rabbit secondary antibody (Promega) were used at 1:5,000 and 1:10,000 dilutions, respectively. The primary antibody against PfalMre11 was generated in rabbit and used at 1:4,000 dilutions, and HRP-conjugated anti-rabbit secondary antibody (Promega) was used at 1:12,000 dilutions. As a control, we used anti-PfHsp70 antibody (kindly provided by Dr. Nirbhay Kumar, Tulane University). We used a PfHsp70 primary antibody at 1:1,000 dilutions and anti-mouse secondary antibody (Promega) at 1:10,000 dilutions. Western blotting was performed as previously described [9]. Proteins were visualized by an enhanced chemiluminescence system (Pierce) and the band intensities were quantified by ImageJ software.

### RNA isolation and RT-PCR

*P*. *falciparum* 3D7 culture having 10% parasitemia was synchronized using 5% sorbitol followed by harvesting at the ring, trophozoite and schizont stages were harvested. Total RNA was isolated from each stage specific culture with and without MMS treatment using the protocol as described [36]. Similarly, total RNA was isolated from yeast strains BSB1-BSB8 and PVY1 after incubation at 30°C using the acid-phenol method as described [33]. Equal amounts of RNA as measured by spectroscopic analysis (JASCO spectrophotometer EMC-709) were subjected to DNase I (Fermentas) digestion to remove contaminating genomic DNA. The absence of genomic DNA was confirmed by PCR prior to the reverse transcription reaction (S1 Fig). Synthesis of cDNA was performed as described [37]. Briefly, ~10 μg of total RNA was reverse transcribed using the reverse transcriptase (Qiagen), and then the cDNA product was subjected to semi-quantitative RT-PCR. Next, the cDNA product was diluted 1:50 and subjected to real time PCR using a SYBR-Green kit (Roche) as described [34] and the Applied Biosystems 7500 Fast Real Time PCR system. The threshold cycle (C_T_) value of *ARP* transcript of each sample was used to normalize the corresponding C_T_ values of *PfalMRE11* transcripts. The normalized C_T_ values of *PfalMRE11* from different samples were compared to each other to obtain ∆C_T_ values. The relative levels of mRNA were deduced from the formula (Change in mRNA level = 2^∆CT^). The mean values (±SD) from three independent experiments were plotted using Graph Pad Prism 6 software. To quantify expression of wild-type and chimeric constructs from the yeast GPD promoter, the C_T_ values were normalized with the C_T_ value of *ScACT1* transcript. All the primers used in the semi-quantitative RT-PCR or real-time RT-PCR are presented in S1 Table.

### MMS sensitivity assay

MMS sensitivity assays were performed as described [34]. Yeast strains BSB1-BSB8 and PVY1 were incubated in media lacking tryptophan at 30°C to OD_600_ 0.8. Half of each culture was spotted on Sc-Trp media containing 0.005% MMS, while the other half of each culture was spotted on Sc-Trp plates without MMS as a control. The plates were incubated for at 30°C for 72 hrs and their growth was compared. Similar experiments were also carried out using YPD media with 0%, 0.005% MMS, or 0.01% MMS.

To quantify the differences in MMS sensitivity of these strains, equal numbers of cells from rapidly dividing cultures were divided them into two groups: one group was treated with 0.005% MMS while the other was untreated. Both groups were incubated for 2 hours at 30°C after which MMS was washed out. Then, equal numbers of MMS-treated and untreated cells were spread onto media lacking tryptophan and incubated at 30°C for 72 hours. To determine the % survival, the ratio of the number of cells grown on MMS to the number of cells grown in the absence of MMS was calculate and multiplied by 100. Each assay was repeated a minimum of 3 times.

### Plasmid end joining assay

Plasmid pRS413-kanMX6 was digested with XhoI and 400 ng of the resulting kanMX6 fragment linear (cut) or 400 ng of the undigested plasmid was transformed into each strain. The ratios of G418-sulphate resistant transformants were calculated for each strain. The assays were repeated thrice and the mean value ± SD was plotted using Graph Pad Prism 6 software.

### Homology modeling

The model of PfalMre11 was obtained by submitting the full-length sequence of PfalMre11 to I-TASSER (Iterative Threading ASSEmbly Refinement) Web Server (http://zhanglab.ccmb.med.umich.edu/I-TASSER/). I-TASSER generates full-length models by a combination of Homology Modeling and Ab-Initio Modeling. For PfalMre11, the system identified and used the 4FBK and 3T1I PDB protein structures as templates that aligned primarily to the nuclease domain of Mre11. The unaligned regions were modeled using Ab-Initio modeling algorithm. We restricted our analysis to the nuclease domain of PfalMre11. Protein structures were derived from the highest confidence model generated by I-TASSER. The PyMOL Molecular Visualization Tool was used to visualize, analyze and generate the images.

## Results

### Identification and primary structure analysis of a Mre11 ortholog in *P*. *falciparum*

**Fig 1:**
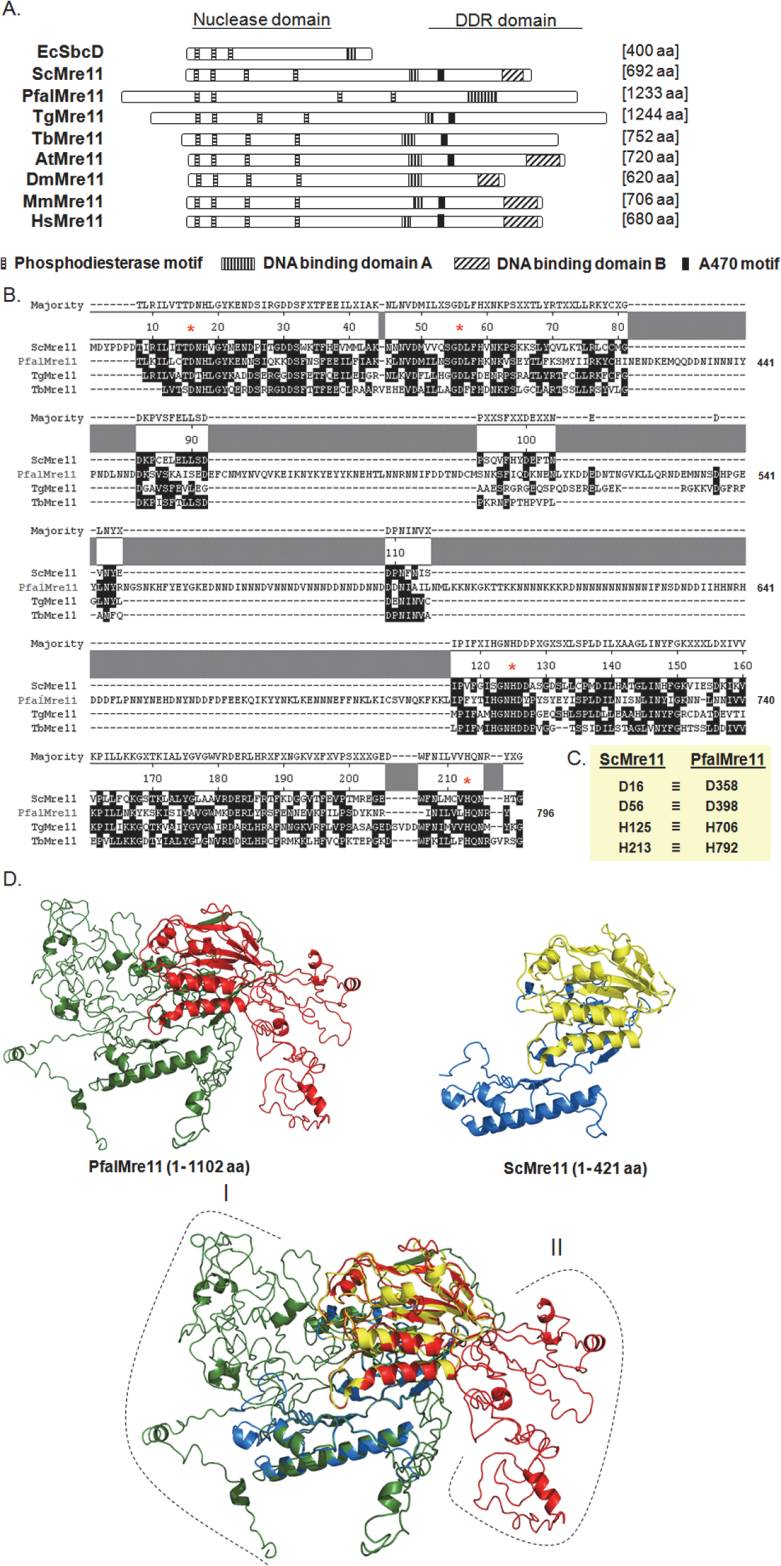
Primary structure analysis of PfalMre11. (A). Schematic representation of different domains and motifs of PfalMre11 in comparison with other Mre11 proteins from bacteria (EcSbcD); yeast (ScMre11); *Toxoplasma gondii* (TgMre11); *Trypanosoma brucei* (TbMre11); *Arabidopsis thaliana* (AtMre11); *Drosophila melanogaster* (DmMre11); mouse (MmMre11) and human (HsMre11). The four phosphodiesterase motifs within the nuclease domain are not only conserved among all the eukaryotic Mre11 but also are present in *E*. *coli* SbcD nuclease protein. PfalMre11 lacks both DNA binding domain-B and A470 motif (AV*(E/K)FV(E/D)K(D/E)(D/E)K*A, where the asterisk refers to sites that accommodate multiple residues). (B) Multiple sequence alignment showing sequence conservation within the nuclease domain of PfalMre11 with ScMre11 and other parasitic Mre11 proteins (TgMre11 and TbMre11). The functionally critical amino-acid residues in each phosphodiesterase motifs (namely, D358, D398, H706 and H792) are marked by red asterisk. The coordinates of the amino-acids positions of ScMre11 is given on the top and that of the PfalMre11 is given on the right. (C) The four critical amino-acid residues of each of the phosphodiesterase motif of ScMre11 (D16, D56, H125 and H213) and the corresponding amino-acids of PfalMre11 are shown. (D) Predicted three-dimensional structure of PfalMre11 N-terminal domain. Homology models of PfalMre11 N-terminal region (amino-acids 1–1102) and ScMre11 N-terminal region (amino-acids 1–421) are shown. The nuclease domain of PfalMre11 is shown in red, while the rest of the N-terminal is shown in green. Similarly, the nuclease domain of ScMre11 is shown in yellow, while the rest of the N-terminal is shown in marine blue. The long N-terminal extension (region I: amino-acids 1–349) and several stretches of insertions within the nuclease domain (region II) of PfalMre11 are also indicated.

A Blast search of PlasmoDB revealed a single copy of the *PfalMRE11* gene in the *P*. *falciparum* 3D7 strain (Gene ID: PF3D7_0107800). The single exon *PfalMRE11* open reading frame (ORF) of 3,699 bp was PCR amplified from genomic DNA. The deduced amino-acid sequence of the PCR product was a 1,233 amino-acid long protein with a molecular mass of 146,149 Da and pI of 5.35. Homology searches of the PfalMre11 protein sequence revealed 14% to 22% overall similarity with other Mre11 orthologs. The PfalMre11 nuclease domain was 33% to 35% identical to the nuclease domains of other Mre11 orthologs (Table 1). Compared to Mre11 orthologs, PfalMre11 had the longest ORF (Fig 1A) and was 80% longer than average (1233 amino acids compared to an average length of 700 amino acids of other Mre11 orthologs). A genome database search of *Toxoplasma gondii*, another apicomplexan parasite, revealed a putative TgMre11 ortholog that was also significantly longer (1244 amino acids) than other Mre11 orthologs (Fig 1A).

**Table 1:** Homology of PfalMre11 with other eukaryotic Mre11 proteins.

	ScMre11	PfalMre11	TgMre11	TbMre11	AtMre11	DmMre11	MmMre11	HsMre11
ScMre11	100	23 (32)	31 (41)	26 (43)	30 (45)	32 (44)	34 (49)	34 (50)
PfalMre11		100	14 (34)	17 (35)	22 (34)	23 (34)	21 (36)	22 (37)
TgMre11			100	27 (45)	32 (42)	31 (43)	35 (47)	35 (46)
TbMre11				100	29 (49)	30 (49)	29 (49)	30 (50)
AtMre11					100	36 (50)	35 (56)	36 (56)
DmMre11						100	41 (57)	39 (56)
MmMre11							100	89 (96)
HsMre11								100

Numbers in parentheses represent homology within the nuclease domain. Sc, *S*. *cerevisiae*, Pfal, *P*. *falciparum*, Tg, *Toxoplasma gondii*, Tb, *Trypanosoma brucei*, At, *Arabidopsis thaliana*, Dm, *Drosophila melanogaster*, Mm, *Mus musculus*, Hs, *Homo sapiens*.

All eukaryotic Mre11 orthologs contain four highly conserved phosphodiesterase motifs in the N-terminal nuclease domain, and the first three are conserved in the EcSbcD, the Mre11 ortholog in *E*. *coli*. Multiple sequence alignments revealed that PfalMre11 also contains the four phosphodiesterase motifs (Fig 1B). Unlike the other Mre11 orthologs, PfalMre11 has a 350 amino acids N-terminal extension before the nuclease domain that extends from amino acid 351–796 (Fig 1A). Such a long N-terminal extension has not been observed in other Mre11 orthologs, with the exception of *T*. *gondii* TgMre11 which has a 220 amino acids N-terminal extension preceding its nuclease domain. The significance of such N-terminal extensions is currently unknown. In yeast Mre11, amino acids that are critical for nuclease function have been identified all four phosphodiesterase motifs [38]. These residues, D16, D56, H125 and H213 of ScMre11) are evolutionary conserved, including in PfalMre11 (D358, D398, H706 and H792) (Fig 1C). In addition, the spacing between the phosphodiesterase motifs is tentatively conserved among all Mre11 orthologs. The spacing between the 1^st^ and the 2^nd^, between the 2^nd^ and the 3^rd^, and between the 3^rd^ and the 4^th^ motifs range from 33–40 amino acids, 69–76 amino acids and 87–99 amino acids, respectively. In PfalMre11 this level of spacing is conserved only between the 1^st^ and the 2^nd^ (40 amino acids) and between the 3^rd^ and the 4^th^ motifs (86 amino acids). There is a large insertion of a low complexity region between the 2^nd^ and the 3^rd^ motifs (308 amino acids) that is not present in other parasitic Mre11 orthologs (i.e., TgMre11 or TbMre11). The significance of such an unusual structural feature within the PfalMre11 nuclease domain remains to be determined.

Mre11 contains two DNA binding domains (DBD): DBD-A is rich in basic amino acids, while DBD-B is rich in acidic amino acids [39, 40]. Although PfalMre11 as well as TgMre11 contain DBD-A, they do not contain DBD-B (Fig 1A). This is consistent with the results of mutational studies in yeast that identified DBD-A as being essential and DBD-B as dispensable for *in vivo* ScMre11 function [40]. At this time, it is difficult to assign any evolutionary significance to the absence of DBD-B in PfalMre11 or TgMre11.

The C-terminal domain of Mre11 plays a crucial role during DDR signaling [18], a function that is conserved in the C-terminal domains of yeast and mammalian Mre11 despite the poor sequence conservation at the amino acid level [41, 42]. The C-terminal domain of PfalMre11 is highly divergent from other Mre11 orthologs, making it impossible to predict whether PfalMre11 also possesses DDR signaling function. Thirteen amino acids, α-helical region specific to Mre11, known as the A470 motif, was first identified in yeast and later found to be conserved in other eukaryotes, with the exception of dipterans [30]. This motif plays regulatory roles in telomere recombination and in telomere rapid deletion [30]. Surprisingly, this motif is missing from PfalMre11, but present in protozoan TgMre11 and TbMre11 (S2 Fig).

Comparison of the predicted three dimensional structures of the N-terminal regions of PfalMre11 and ScMre11 by homology modeling revealed striking structural similarity in their nuclease domains despite having a relatively low level of sequence identity (32%). Indeed, it is a near perfect superimposition of the core nuclease domains. The superimposed image revealed that the long N-terminal extension (region I) and the low complexity insertion (region II) protrude out of the core structure (Fig 1D).

### Expression of recombinant PfalMre11 protein and generation of antibody

The *PfalMRE11* ORF was cloned into three bacterial expression vectors (pET28a, pGEX and pMALC2) and the recombinant plasmids were each transformed into three strains of *E*. *coli* (namely, BLl21 DE3, Rosetta DE3 and BL21 pLysS DE3). Western blot analyses using anti-His antibody confirmed that recombinant PfalMre11 was not expressed (data not shown). The PfalMre11 N-terminus (PfalMre11-N: 832 amino acids) and the PfalMre11 C-terminus (PfalMre11-C: 377 amino acids) were separately cloned into the pET28a vector and transformed into the *E*. *coli* strains described above. Only the expression of PfalMre11-C protein was observed in the Rosetta DE3 strain (having the pRARE plasmid that supplies tRNAs for six rare amino acids). S3A Fig (lane 5) shows that recombinant PfalMre11-C corresponds to about 15% of the total protein in the crude cell extract after IPTG induction. Expression of the recombinant protein was confirmed by Western blot analysis (S3B Fig). Because the induced recombinant protein was predominantly expressed in the insoluble fraction and could not be purified; therefore, the induced protein band was excised from the SDS-PAGE gel, purified and and injected into rabbits for antibody production. Rabbit anti- PfalMre11 antibody reacted positively with recombinant PfalMre11-C protein expressed in bacteria, confirming the specificity of the antibody (S3C Fig). When tested with parasite lysate, the anti-PfalMre11 antibody specifically recognized a protein band near 145 kDa that was not detected by pre-immune sera (S3D Fig). The size of the band corresponded to the predicted molecular mass of the PfalMre11 protein (146 kDa).

### Developmentally regulated expression of PfalMre11 during blood stage development

**Fig 2:**
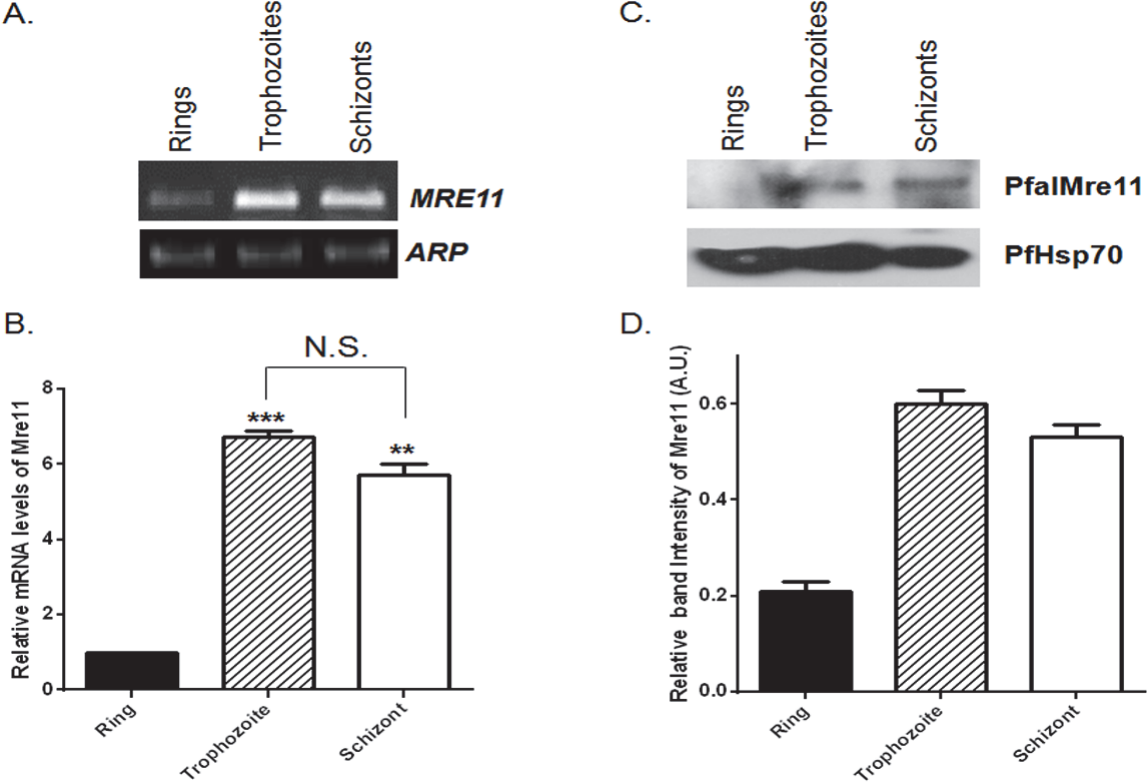
Developmentally regulated expression of PfalMre11 during blood stage development. (A). Semi-quantitative RT-PCR showing expression of *PfalMRE11* mRNA at the ring, trophozoite and schizont stages. *PfARP* was used as the loading control. (B). Relative abundance of *PfalMRE11* transcript measured by real-time RT-PCR analysis. Data were normalized against *PfARP*. The mean values ± SD from three independent experiments are plotted. (C). Stage specific expression of PfalMre11 protein. The stages are marked on the top. PfHsp70 was used as the loading control. (D). The quantification of Western blots from three independent experiments. Data was normalized against the loading control PfHsp70. Each bar represents mean density ± SD. The *P* value was calculated using the two-tailed Student’s t-test (** means *P* <0.01; *** means *P* <0.001; N.S. means not significant).

To examine the expression of *PfalMRE11* during the intra-erythrocytic growth of *P*. *falciparum*, we synchronized parasites at the ring, trophozoite and schizont stages and performed RT-PCR and Western blot analysis. Semi-quantitative RT-PCR data showed that the steady-state levels of the *PfalMER11* transcripts were more abundant in the trophozoite and schizont stages than in the ring stage (Fig 2A). Expression of another parasitic gene *PfARP* (Asparagine Rich Protein) remained unchanged throughout intra-erythrocytic development and hence was used as the loading control. To more accurately estimate levels of *PfalMRE11* transcripts during the three growth stages, we performed real-time RT-PCR and normalized the data with the expression levels of the *PfARP* transcripts. Compared to the ring stage, the PfalMRE11 transcript was 7 times more abundant in the trophozoite stage and 5 times more abundant in schizont stage (Fig 2B). Stage-specific expression of PfalMre11 protein was consistent with the RT-PCR results (Fig 2C). PfHsp70 protein levels remained stable during all three parasitic developmental stages and served as the loading control. Quantification of the band intensities revealed that the steady-state levels of PfalMre11 protein were 3 times and 2.5 times more abundant in the trophozoite and schizont stages, respectively, than in the ring stage (Fig 2D). Thus, the stage-specific changes in expression of PfalMRE11 at the RNA and the protein levels demonstrate that it is developmentally regulated and that such regulation is more likely to occur at the level of transcription rather than translation.

### DNA damage- induced up-regulation of PfalMre11 during intra-erythrocytic development

**Fig 3:**
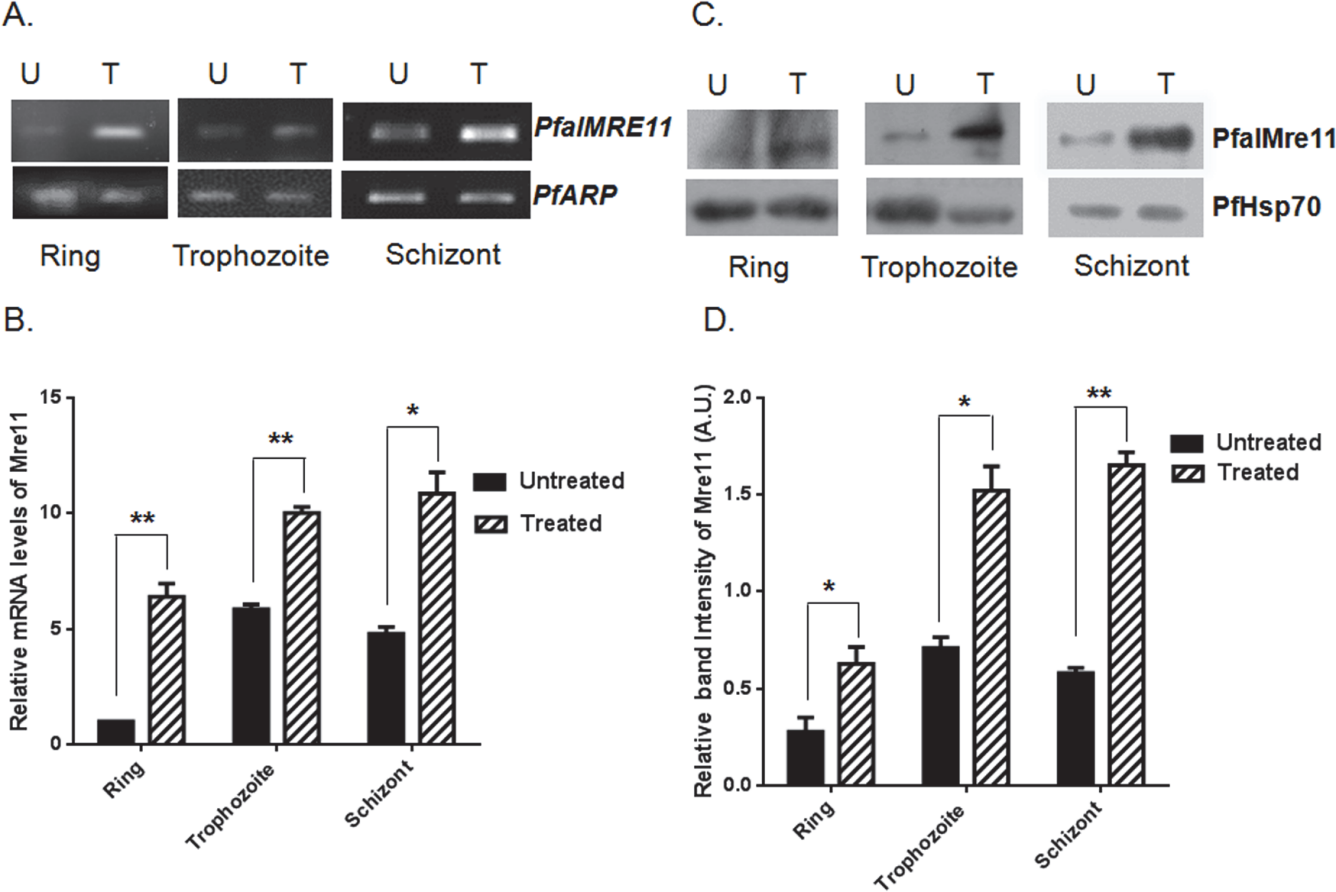
DNA damage induced up-regulation of PfalMre11 during intra-erythrocytic development. (A) *P*. *falciparum in vitro* cultures were synchronized at the ring, trophozoite or schizont stage and then either un-treated (U) or treated with 0.05% MMS (T) for six hours. Semi-quantitative RT-PCR from extracted RNA revealed up-regulation of *PfalMRE11* mRNA at all stages. *PfARP* transcript was used as the loading control. (B). Real-time RT-PCR data showing fold up-regulation of *PfalMRE11* mRNA at the ring, trophozoite and schizont stage, respectively. Data were normalized with the abundance of *PfARP* transcript. Each bar represents mean value ± SD from three independent experiments. (C). Western blots showing MMS induced expression of PfalMre11 protein at the ring, trophozoite and schizont stages. U: untreated; T: treated with MMS. PfHsp70 acted as the loading control. (D). The quantification of Western blots exhibiting fold induction of PfalMre11 at the ring, trophozoite and schizont stages. Data were normalized against the loading control PfHsp70. Each bar represents mean density ± SD from three independent experiments. The *P* value was calculated using the two-tailed Student’s t-test (* means *P* <0.05; and ** means *P* <0.01).

Previous studies have shown that *MRE11* expression isup-regulated in response to DNA damage in a variety of organisms [43–45]. Mre11 is a “first responder” to DSBs where it activates the DDR pathway and recruits various effector molecules involved in DNA repair mechanisms [46]. We sought to investigate: (a) whether *PfalMRE11* is up-regulated in response to genome-wide DNA damage caused by MMS, and (b) whether such up-regulation is developmentally regulated. To this end, we synchronized *P*. *falciparum* at the ring, trophozoite and schizont stages, treated the cultures with MMS, and compared *PfalMRE11* transcript levels in both treated and untreated cultures using semi-quantitative RT-PCR and real-time RT-PCR analyses. *PfalMRE11* transcripts were up-regulated in response to MMS treatment at all three developmental stages (Fig 3A). Real-time RT-PCR data revealed that upon DNA damage, *PfalMRE11* expression was 6-fold greater at the ring stage, and 2-fold greater at the trophozoite and schizont stages, than *PfalMRE11* expression in untreated cultures (Fig 3B). Equal amounts of total RNA from untreated and treated cultures were used in these experiments. The expression of house-keeping gene *PfARP* remained unaffected upon MMS treatment and hence it is used as the normalization control.

Next, PfalMre11 protein expression was examined in cultures that were synchronized at the ring, trophozite and schizont stages and treated with MMS. At 6h of MMS treatment, expression of PfalMre11 was markedly increased compared to untreated cultures at all three developmental stages (Fig 3C). Quantification of the band intensities showed nearly 2-fold increase in PfalMre11 expression at all the three stages (Fig 3D).

### Functional complementation of a *∆mre11* mutant of *S*. *cerevisiae* by PfalMre11 nuclease domain in conjunction with the DDR domain of ScMre11

To confirm *PfalMRE11* as a bona fide *MRE11* ortholog, we performed a functional complementation assay to determine if *PfalMRE11* could functionally complement a yeast *∆mre11* null mutant. The strains BSB3, BSB2 and BSB8 were constructed by transforming pTA*PfalMRE11*, pTA*ScMRE11* and pEmpty plasmids into the *∆mre11* strain, respectively. Since *∆mre11* cells are hyper-sensitive to MMS, a rescue of the MMS sensitivity phenotype would indicate complementation of Mre11 function. We observed no functional complementation of *∆mre11* by *PfalMRE11* (data not shown).

**Fig 4:**
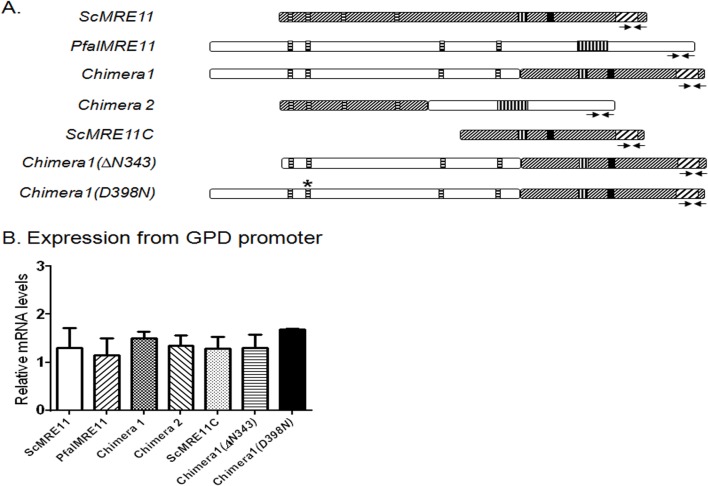
Expression levels of different constructs used in functional complementation. (A). Schematic representations of genes or gene fragments used in the complementation experiments. ScMre11: full length *MRE11* gene from *S*. *cerevisiae*. *PfalMRE11*: full length *MRE11* gene from *P*. *falciparum*. Chimera 1: chimeric gene consisting of DNA sequences corresponding to the N-terminal of PfalMre11 (1–832 amino acids) fused to the DNA sequence corresponding to the C-terminal domain of ScMre11 (315–692 amino-acids). Chimera 2: chimeric gene consisting of DNA sequences corresponding to the N-terminal of ScMre11 (1–256 amino acids) fused to the DNA sequence corresponding to the C-terminal domain of PfalMre11 (828–1233 amino-acids). *ScMRE11C*: gene fragment corresponding to the C-terminal domain of ScMre11 (315–692 amino-acids). Chimera 1(∆N343): chimeric gene consisting of DNA sequences corresponding to PfalMre11 (344–832 amino acids) fused to the DNA sequence corresponding to the C-terminal domain of ScMre11 (315–692 amino-acids). Chimera 1 (D398N): same as chimera 1 with a missense mutation (D to N) at the 398^th^ amino-acid of PfalMre11. (B). Real-time RT-PCR data showing comparable expression of each gene or gene fragment used in the complementation assay. In each case mean value (± SD) from three different experiments is normalized against the abundance of *ACT1* mRNA.

ScMre11 has two distinct activities that are required for repair of DSB: the N-terminally confined nuclease activity and the C-terminally located DDR activity. As demonstrated above, the N-terminus of PfalMre11 shares considerable sequence identity with other Mre11 orthologs, and the PfalMre11 nuclease domain shares structural similarity with ScMre11. On the other hand, the C-terminus of PfalMre11 is highly divergent compared to other Mre11 orthologs and shares limited sequence similarity with ScMre11. Therefore, we hypothesized that PfalMre11 possessed N-terminal nuclease activity, and the C-terminal domain could not interact with molecules involved in the yeast DDR pathway. To test this hypothesis, we constructed several complementation vectors carrying recombinant genes (Fig 4A) and transformed then independently into a yeast *Δmre11* strain to determine if the N-terminus or C-terminus of PfalMre11 could complement Mre11 function. Chimera 1 consisted of the N-terminal region of PfalMre11 (amino acids 1–832) and the C-terminal region of ScMre11 (amino acids 315–692); Chimera 2 consisted of the N-terminal domain of ScMre11 (amino acids 1–256) and the C-terminal region of PfalMre11 (amino acids 727–1132). Another complementation vector was constructed with the C-terminal region of ScMre11 alone. These regions of *PfalMRE11* and *ScMRE11* genes were selected based on homology analysis of the nuclease domain. Expression levels of the hybrid genes were confirmed by real-time RT-PCR (Fig 4B); however, the proteins could not be detected by Western blot analysis using anti-Myc antibody, possibly due to low expression levels.

**Fig 5:**
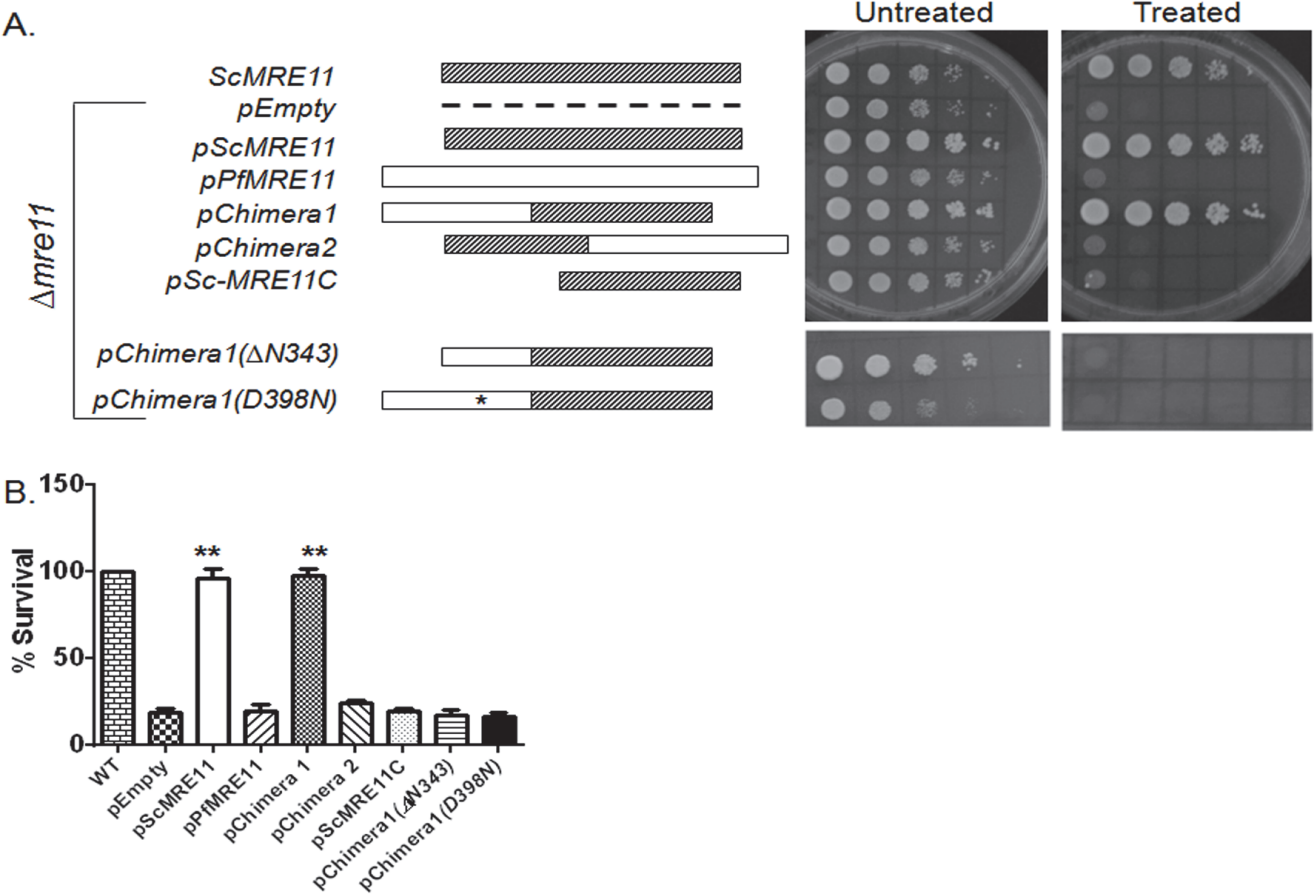
Functional complementation of *∆mre11* mutant of *S*. *cerevisiae* by PfalMre11 nuclease domain in conjunction with the DDR domain of ScMre11. (A). Spotting assay on media lacking tryptophan without (untreated) or with (treated) 0.005% MMS supplementation. The relevant genotypes along with the corresponding schematic diagrams are shown on the left. (B) Various complementation strains (as shown on the X-axis) were treated with 0.005% MMS for 2 hours and then returned to growth on media lacking tryptophan and MMS. The number of surviving colonies were scored and compared with the number arising from un-treated cells. Each bar represents the mean number ±SD after normalizing with untreated controls from four independent experiments. The *P* value was calculated by two-tailed Student’s t-test (** indicates *P* <0.01).

Next, *∆mre11* strains harboring different complementation plasmids were tested for functional complementation analysis. We examined MMS sensitivity using a spotting assay and a return-to-growth assay. In the spotting assay, dilutions of different strains were spotted on MMS containing plates and allowed to grow for several days. Thus, the cells were continuously exposed to MMS for the entire growth period. On the other hand, in the return-to-growth assay, the cells were exposed to MMS for two hours and then returned to grow on plates lacking MMS. Thus, the first experiment tests the cells’ ability to survive when challenged with continuous DNA insult and the second experiment measures the cells’ ability to repair DNA damage after a brief exposure to MMS. Chimera 1 rescued MMS sensitivity of *∆mre11* which is comparable to ScMre11 (Fig 5A and S4 Fig). Chimera 2 remained as hyper-sensitive to MMS as the *∆mre11* null strain (Fig 5A). Since Chimera 1 contained the C-terminal domain of ScMre11, we also tested the possibility that the reversal of MMS sensitivity was not due solely to the C-terminal domain of ScMre11. When the C-terminal domain of ScMre11 was expressed alone, we found no reversal of MMS sensitivity (Fig 5A). Taken together, these results support the hypothesis that the N-terminus of PfalMre11 has nuclease function, but the C-terminus lacks the DDR function ascribed to ScMre11.

To gain further insights into the structure-function relationship of the PfalMre11 N-terminal domain, we generated two mutants of Chimera 1: a missense mutant (D398N) and a deletion mutant (∆1–343). It was previously reported that aspartic acid (D) at the 56^th^ position of ScMre11 lies in the second phosphodiesterase motif and is critical for nuclease activity [47]. Using site-directed mutagenesis, we created a corresponding amino acid substitution, D398N, in Chimera 1. When tested for functional complementation, this mutant exhibited *∆mre11* like MMS hyper-sensitivity (Fig 5A), suggesting that PfalMre11^D398N^ is also a nuclease-deficient mutant. Such a dramatic effect of D398N is surprising, given that the corresponding yeast mutant *mre11(D56N)* is only mildly sensitive to MMS (S4 Fig) [47]. As stated earlier, the PfalMre11 nuclease domain covers amino acids 350–796 and is preceded by an unstructured N-terminal extension which is absent in all other Mre11 orthologs. In order to understand whether this unusual N-terminal extension is dispensable for the N-terminal function of PfalMre11, we deleted the first 343 amino acids of Chimera 1 and tested the construct for MMS sensitivity in a *mre11* null yeast strain. This deletion mutant failed to complement loss of ScMre11 function to normal levels, suggesting that the entire N-terminal extension of PfalMre11 is indispensable for the N-terminal function of PfalMre11 (Fig 5A and S4 Fig). It is possible that only a section of this 343 amino acid stretch is indispensable; however, since this region does not contain any particular motif/structural elements, it is difficult to design smaller deletion mutants to test this possibility. The expression levels of both the D398N and N-terminal deletion mutants were tested by real-time RT-PCR analysis and mRNA expression of both of the mutants were on par with the mRNA expression of Chimera 1 (Fig 4B).

Return to growth experiments also revealed similar results. Chimera 1 complemented ScMre11 deficiency, while the phenotype observed with either PfalMre11 or Chimera 2 was indistinguishable from the *∆mre11* null strain (Fig 5B). This result suggests that the N-terminal domain of PfalMre11 in conjunction with the C-terminal domain of ScMre11 is capable of repairing MMS induced DNA breaks. Such repair activity was abrogated by Pfal D398N and PfalMre1-N-terminal (∆343) also abrogated the repair activity of Chimera 1 (Fig 5B). Since the proteins could not be detected by Western blot, we cannot formally rule out the possibility that the lack of complementation is not due to reduced stability of the recombinant proteins.

### PfalMre11 is capable of interacting with ScRad50 and ScXrs2

**Fig 6:**
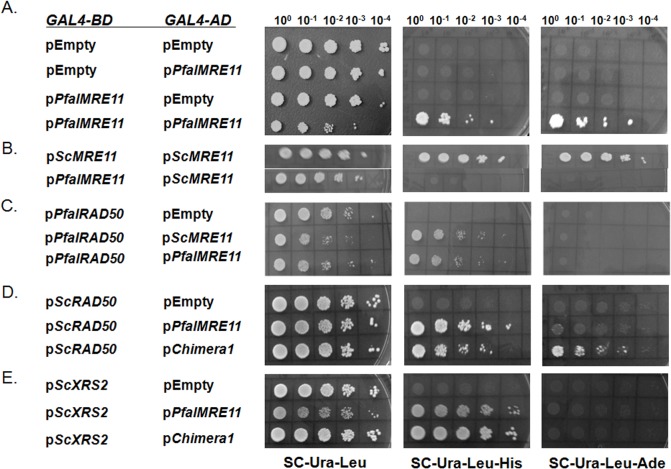
PfalMre11 is capable of forming MRX complex in yeast. (A) A DNA fragment corresponding to the full length *PfalMRE11* ORF was fused to the *GAL4* activation domain (*GAL4-AD*) in pGADC1 as well as to the *GAL 4* DNA binding domain (*GAL-BD*) in pGBDUC1. Two hybrid interactions were tested with yeast strain PJ694A, which carries *ADE2* and *HIS3* genes as reporters. Starting with the same OD (1 OD/ml), five fold serial dilutions were prepared and spotted on media lacking uracil and leucine (SC-Ura-Leu) as a control, as well as media lacking uracil, leucine and histidine (SC-Ura-Leu-His) or media lacking uracil, leucine and adenine (SC-Ura-Leu-Ade) to test for protein-protein interactions. (B) *ScMRE11* or *PfalMRE11* were cloned into pGBDUC1 to produce fusion protein with *GAL4* DNA binding domain. Interactions with *ScMRE11* were scored. (C) *PfalRAD50* were cloned into pGBDUC1 to produce fusion protein with *GAL4* DNA binding domain. Interactions with *ScMRE11* or *PfalMRE11* were scored. (D) *ScRAD50* was cloned into pGBDUC1 to produce a fusion protein with a *GAL4* DNA binding domain. Interactions with *PfalMRE11* or Chimera 1 were scored. (E) *ScXRS2* was fused to the DNA binding domain in pGBDUC1 and tested for interaction with *PfalMRE11* or Chimera 1.

Mre11 acts as MRX complex in yeast, where two molecules of Mre11, two molecules of Rad50 and one molecule of Xrs2 form a hetero-pentameric complex. The demonstration that Chimera 1 (N-terminus of PfalMre11 fused to C-terminal of ScMre11) could sufficiently complement ScMre11 deficiency suggests that it interacts in a MRX complex consisting of Chimera1-ScRad50-ScXrs2. We used a yeast two hybrid system to test whether PfalMre11 (or Chimera 1) could interact with itself as well as ScRad50 and ScXrs2. A *GAL4* transcriptional activation system in PJ69-4A cells was used to distinguish robust and feeble interactions between bait and prey proteins; a robust interaction was indicated by growth on (Sc-Leu-Ura-Ade) triple dropout plates and a weaker interaction was indicated by growth on (Sc-Leu-Ura-His) triple dropout plates. We observed robust self-interaction of PfalMre11 protein (Fig 6A). These results suggest that PfalMre11 may act as a dimer, consistent with other eukaryotes. Interaction between full length PfalMre11 and full length ScMre11 was not observed (Fig 6B). We also observed physical interaction between PfalMre11 and PfalRad50, suggesting the existence of the MR complex in the parasite (Fig 6C). When tested for interaction with ScRad50, both PfalMre11 and Chimera 1 interacted; however, the interaction between PfalMre11 and ScRad50 was significantly weaker (Fig 6D). PfalMre11 as well as Chimera 1 also interacted with ScXrs2; however, these interactions were not robust given that growth was observed only on (Sc-Leu-Ura-His) triple dropout plates (Fig 6E). Taken together, these results suggest that PfalMre11 interacts with individual components of the MRX complex in the heterologous yeast system.

### NHEJ activity of Chimera 1

**Fig 7:**
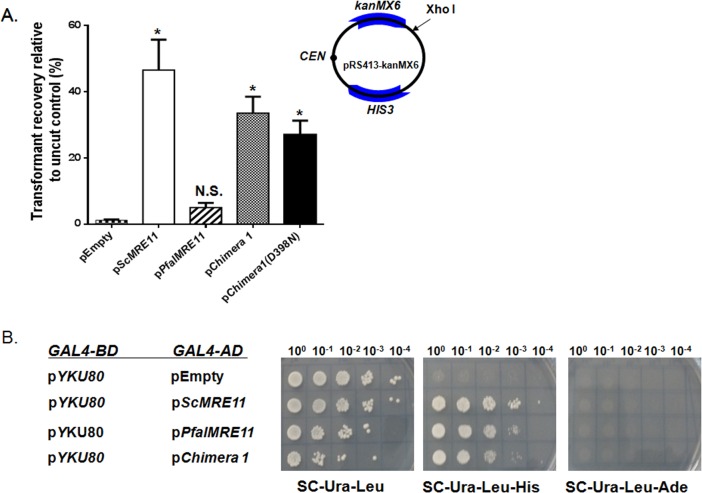
End joining activity of Chimera 1 consisting of PfalMre11 nuclease domain in conjunction with the DDR domain of ScMre11. (A) The relevant genotype of each strain is marked on the X-axis. The schematic diagram of the plasmid used for this assay is shown in the inset. The end joining assays were performed as described in the Method section. (B) PfalMre11 is capable of interacting with YKu80. Yeast-two- hybrid analysis between YKu80 cloned into the bait vector (pGBDUC1) vector and PfalMre11 or Chimera 1 cloned into the prey vector (pGADC1). ScMre11 acted as a positive control. The *P* value was calculated by two-tailed Student’s t-test (* indicates *P* <0.05, N.S. means not significant).

Mre11 plays crucial role in NHEJ mediated DSB repair. Since our results demonstrated that PfalMre11 repairs MMS-induced DNA damage, and interacts with ScRad50 and ScXrs2, we wanted to investigate whether PfalMre11 acts in in DSB repair through NHEJ. To test this, we used a plasmid transformation assay in which the efficiency of the recircularization of a linearized plasmid was used as a measure of NHEJ (Fig 7A). Yeast strains harboring different complementation constructs were transformed with an equal amount of either linear plasmid or circular plasmid (control). The efficiency of NHEJ was measured as the ratio of the number of transformants obtained with linear plasmid to the number of transformants obtained with circular plasmid. Compared to the control, Chimera 1 showed moderate NHEJ efficiency compared to ScMre11 while the relatively low number of transformants with PfalMre11 indicated no significant NHEJ activity. The Chimera 1 (D398N) mutant did not resulted in abrogation of NHEJ activity (Fig 7A), which corroborates with the previous finding that the nuclease activity of ScMre11 is dispensable for NHEJ activity [47]. A large body of evidence has demonstrated that interaction between Mre11 and yKu80 is important for NHEJ [48]; therefore, we investigated whether Chimera 1 or PfalMre11 also interacts with yKu80. We observed that both Chimera 1 and PfalMre11 interacted with yKu80, although the interactions were not robust (Fig 7B). Since the Ku complex as well as NHEJ are apparently absent in *P*. *falciparum*, the physiological significance of these findings is currently uncertain.

## Discussion

Here we provide several lines of evidence that show the ORF (Gene ID: PF3D7_0107800), described as a putative Mre11 ortholog in PlasmoDB, codes for a bona fide Mre11 protein in *P*. *falciparum*. Firstly, this protein is predominantly expressed during the mitotically active schizont stage of the parasite. Secondly, the expression of PfalMre11 is induced in response to DNA damage. Thirdly and most importantly, the nuclease domain of PfalMre11 can functionally complement the nuclease function of yeast Mre11. Fourthly, PfalMre11 can interact with PfalRad50. Finally, PfalMre11 is also capable of forming a complex with yeast Rad50 and Xrs2, two factors that are essential for Mre11 activity.

Although, the steady-state level of PfalMre11 was less abundant at the ring stage as compared to the trophozoite or the schizont stage, its up-regulation in response to DNA damage was observed at all three stages. This finding implies that the regulatory trans-factors that activate PfalMRE11 gene expression upon DNA damage are likely to be present at all stages during intra-erythrocytic development in the host. Given that the trophozoite and the schizont stages are metabolically more active and hence are likely to be more prone to DNA damaging free radicals, the question arises whether the increased abundance of PfalMre11 at these later stages is in response to spontaneous DNA damage as a result of exposure to free radicals or replication stress, or is purely developmentally regulated. Currently, the answers to such questions are unknown.

In eukaryotes, a DNA DSB is repaired either by the HR or NHEJ pathway. Mre11 plays a central role during the “decision-making” process that leads to either HR or NHEJ. Although *Plasmodium* apparently lacks NHEJ, it does possess the poorly defined A-NHEJ pathway [8]. It is not known whether PfalMre11 plays a similar role during the pathway choice of either HR or A-NHEJ. Given that *Plasmodium* has a haploid genome, a DNA DSB can be repaired by the HR mechanism only during or after the S phase (the trophozoite and the schizont stages). A DSB occurring in the G1 phase (the ring stage) is likely to be repaired by another mechanism, e.g. A-NHEJ. Currently, it is not known to what extent, if at all, A-NHEJ pathway is utilized in the ring stage of *P*. *falciparum*. Nonetheless, since Mre11 is common to all eukaryotic DSB repair pathways, it is not surprising that PfalMre11 can be found at all cell cycle phases in *Plasmodium*.

The DDR function of MRX is mediated through the interaction of Xrs2 (Nbs1) with Tel1 (the yeast ortholog of *ATM*) [49]. The lack of PfXrs2/Nbs1 and *PfATM* or *PfATR* in the *Plasmodium* genome sequences is puzzling. It is possible that *Plasmodium* possesses the structural and functional orthologs of these important DDR proteins but the amino acid sequences of these proteins may not be conserved. Our finding that PfalMre11 is capable of interacting with ScXrs2 supports this notion. Although, PfalRad50 has been annotated in the *Plasmodium* genome, earlier work on genome-wide yeast two hybrid analysis using a cDNA prey library of 400–500 bp average size failed to identify interaction between PfalMre11 and PfalRad50 [50]. In the current study, we are able to detect weak interaction between PfalMre11 and PfalRad50 using a full length genomic fragment of the *PfalRAD50* ORF. This finding once again suggests the conservation of PfalMre11 function.

Our results demonstrated that PfalMre11 failed to functionally complement ScMre11 in an *mre11* null mutant, whereas a fusion between the nuclease domain of PfalMre11 and the DDR domain of ScMre11 successfully complemented *∆mre11*. We also demonstrated that both the fusion protein (Chimera 1) and the PfalMre11 can form the MRX complex in yeast. Thus, we speculate that other important yeast proteins of the DDR pathway which interact with ScMre11 could not interact with full length PfalMre11, but they could interact with the Chimera 1 protein, resulting in functional complementation. Additional studies are needed to shed light on the nature of these interactions. We observed that the Chimera 1 (D398N) nuclease mutant is defective in repairing MMS induced DNA damages but it could engage in plasmid end joining, suggesting that the nuclease activity of Chimera 1 is dispensable for NHEJ. Currently, we do not know why this mutant displayed such a strong MMS sensitive phenotype while the corresponding yeast mutant (D56N) is only mildly sensitive to MMS. It could be possible that this mutation affects the structure of Chimera 1 in such a way that the protein fails to engage itself with other DNA repair proteins involved in this pathway.

The 13 amino-acid alpha-helical region of ScMre11 (known as the A470 motif) is evolutionarily conserved from yeast to humans, with the exceptions of dipterans and, as reported here, *Plasmodium*. This motif is involved in several aspects of telomere maintenance, including telomerase-mediated elongation and telomere repeat recombination. Given that telomere maintenance in dipterans is transposition based, one possible interpretation for absence of the Mre11 A470 motif is because it is specific to the telomerase pathway and telomere repeat recombination. However, *P*. *falciparum* lacks the A470 motif although it is dependent on telomerase-mediated telomere addition. The role of PfalMre11 in telomere maintenance remains an open question.

It is well established that loss of Mre11 function in both yeast and humans causes sensitivity to DNA damage [51, 52]. Since Mre11 is involved in the upstream DDR pathway, which is common to all eukaryotic DSB repair mechanisms, it is likely that inactivation of PfalMre11 function could render the malaria parasites vulnerable to DNA damage. Since *Plasmodium* and human Mre11 proteins share only 22% sequence identity, it might be possible to specifically target the parasitic Mre11 with small molecule inhibitors.

## Supporting Information

S1 Fig (A) Minus RT-PCR on DNase treated RNA isolated from different yeast strains (as indicated on the top) shows absence of genomic DNA contamination in the samples. Primer pair OSB14 and OSB16 was used to amplify a 307 bp fragment of the *ACT1* gene/mRNA. PCR was performed for 30 cycles. (B) Minus RT-PCR on DNase treated RNA isolated from different stages *of P*. *falciparum in vitro* cultures shows absence of genomic DNA contamination. Primer pair OSB94, OSB95 was used to amplify a 300 bp fragment of the *PfARP* gene/mRNA. (C) Minus RT-PCR on DNase treated RNA isolated from different stages of untreated (control) or MMS treated (MMS) *P*. *falciparum in vitro* cultures showed absence of genomic DNA contamination. Primer pair OSB94, OSB95 was used to amplify a 300 bp fragment of the *PfARP* gene/ mRNA. M: DNA molecular weight marker (as marked on the left).
(TIF)

S2 Fig Multiple sequence alignment reveals the lack of A470 motif in PfalMre11. The coordinate of ScMre11 and PfalMre11 amino-acid sequences are given on the left. The highly conserved amino-acid residues are highlighted in cyan and the semi-conserved residues are highlighted in pink.
(TIF)

S3 Fig Expression of recombinant PfalMre11 protein and generation of antibody. (A). A polypeptide corresponding to the last 377 amino-acids of PfalMre11 (as indicated by the hatched box) was expressed as an N-terminal His-tagged protein (marked by an arrow). Expression profile from cell lysates taken at 0 hour (lane 1); un-induced for 4 hour (lane 4) and induced with IPTG for 4 hours (lane 5) are shown. Lanes 2 and 3 represent protein profile from cells carrying empty vector and induced with IPTG for 4 hours or un-induced for 4 hours, respectively. Protein molecular weight markers (M) are indicated on the left side. (B). The expressed recombinant protein is recognized by anti-His antibody. Lane 1: empty vector; and lane 2: induced sample. (C). The anti-PfalMre11 antibody generated against the recombinant PfalMre11 protein (377 amino-acids at the C-terminal region) recognizes the recombinant protein from bacterial lysates. Lane 1: empty vector; and lane 2: induced sample. (D). The anti-PfalMre11 antibody detects a parasite protein of molecular weight 145 kDa (indicated by an arrow). PI: pre-immune sera; I: immune sera.
(TIF)

S4 Fig Chimera 1 ^D398N^ mutant exhibits much stronger MMS sensitive phenotype than the corresponding yeast mutant ScMre11^D56N^. Spotting assays on YPD media without (untreated) or with (treated) 0.005% or 0.01% MMS supplementation are shown. The relevant genotypes are shown on the left.
(TIF)

S1 Table Primers used in this study. (DOC)

S2 Table Yeast strains used in this study. (DOC)
