# Correction: Identification of *Plasmodium falciparum* DNA Repair Protein Mre11 with an Evolutionarily Conserved Nuclease Function

**DOI:** 10.1371/journal.pone.0293936

**Published:** 2023-10-31

**Authors:** Sugith Babu Badugu, Shaik Abdul Nabi, Pratap Vaidyam, Shyamasree Laskar, Sunanda Bhattacharyya, Mrinal Kanti Bhattacharyya

The third author’s name is spelled incorrectly. The correct name is: Pratap Vydyam.

The correct citation is: Badugu SB, Nabi SA, Vydyam P, Laskar S, Bhattacharyya S, Bhattacharyya MK (2015) Identification of Plasmodium falciparum DNA Repair Protein Mre11 with an Evolutionarily Conserved Nuclease Function. PloS ONE 10(5): e0125358. https://doi.org/10.1371/journal.pone.0125358
