# Supplementary figures and images for: Identification of Plasmodium falciparum DNA Repair Protein Mre11 with an Evolutionarily Conserved Nuclease Function
Source: PLoS One. 2015 May 4;10(5):e0125358. doi: 10.1371/journal.pone.0125358 (PMC4418825; doi:10.1371/journal.pone.0125358)

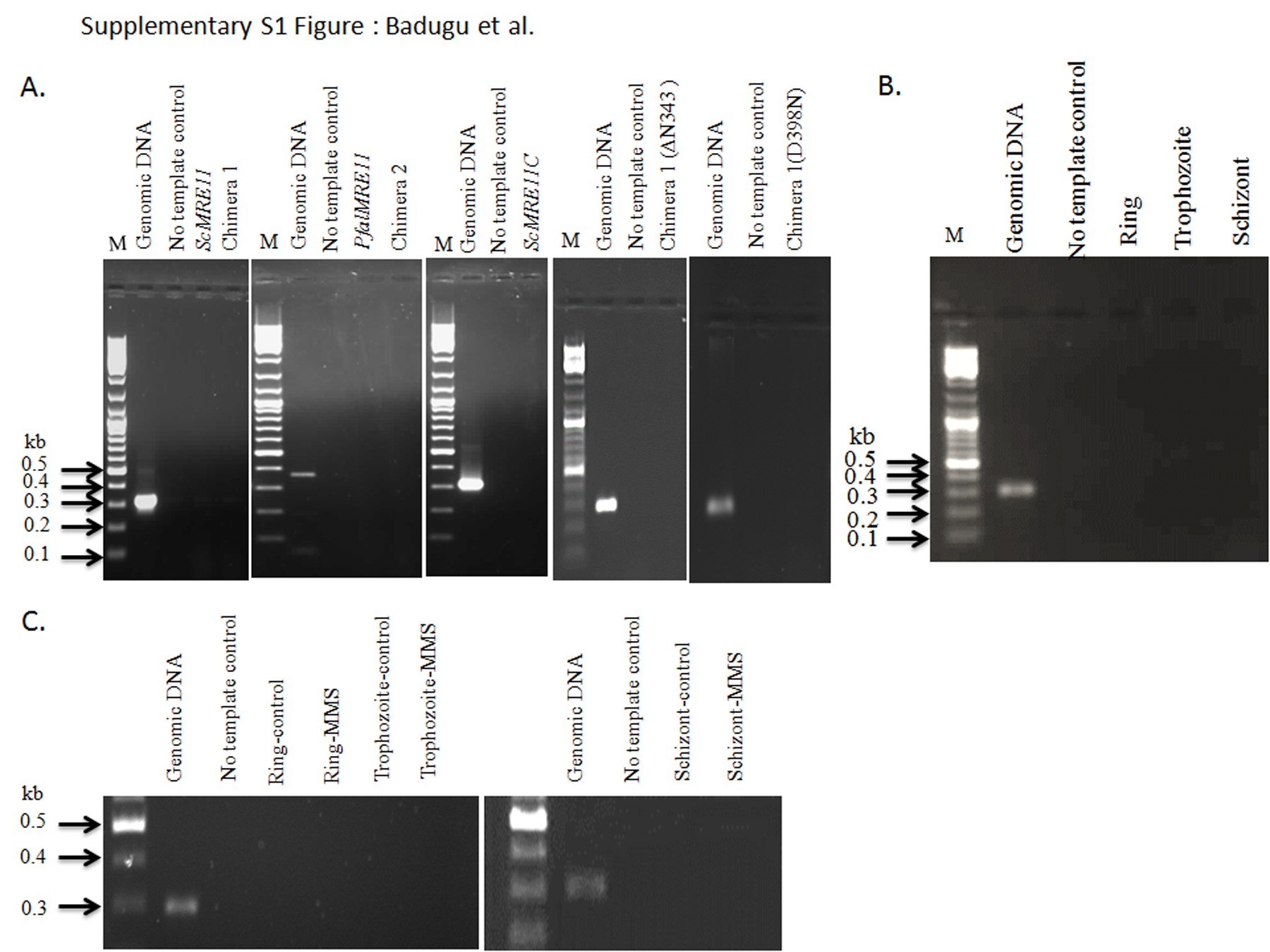

Supplement: S1 Fig — Primer pair OSB14 and OSB16 was used to amplify a 307 bp fragment of the ACT1 gene/mRNA. PCR was performed for 30 cycles. (B) Minus RT-PCR on DNase treated RNA isolated from different stages of P. falciparum in vitro cultures shows absence of genomic DNA contamination. Primer pair OSB94, OSB95 was used to amplify a 300 bp fragment of the PfARP gene/mRNA. (C) Minus RT-PCR on DNase treated RNA isolated from different stages of untreated (control) or MMS treated (MMS) P. falciparum in vitro cultures showed absence of genomic DNA contamination. Primer pair OSB94, OSB95 was used to amplify a 300 bp fragment of the PfARP gene/ mRNA. M: DNA molecular weight marker (as marked on the left). (TIF) [file pone.0125358.s001.tif]

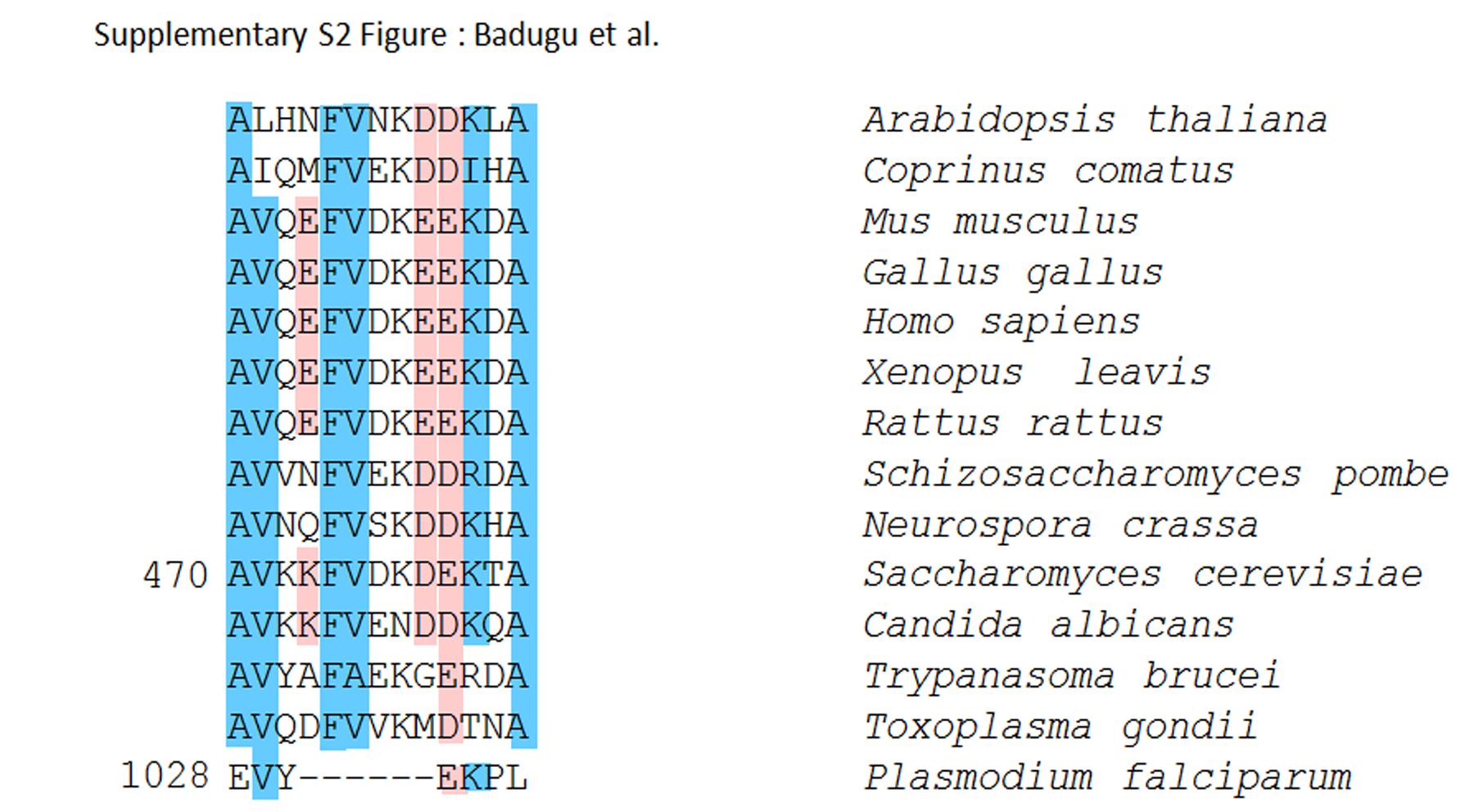

Supplement: S2 Fig — The coordinate of ScMre11 and PfalMre11 amino-acid sequences are given on the left. The highly conserved amino-acid residues are highlighted in cyan and the semi-conserved residues are highlighted in pink. (TIF) [file pone.0125358.s002.tif]

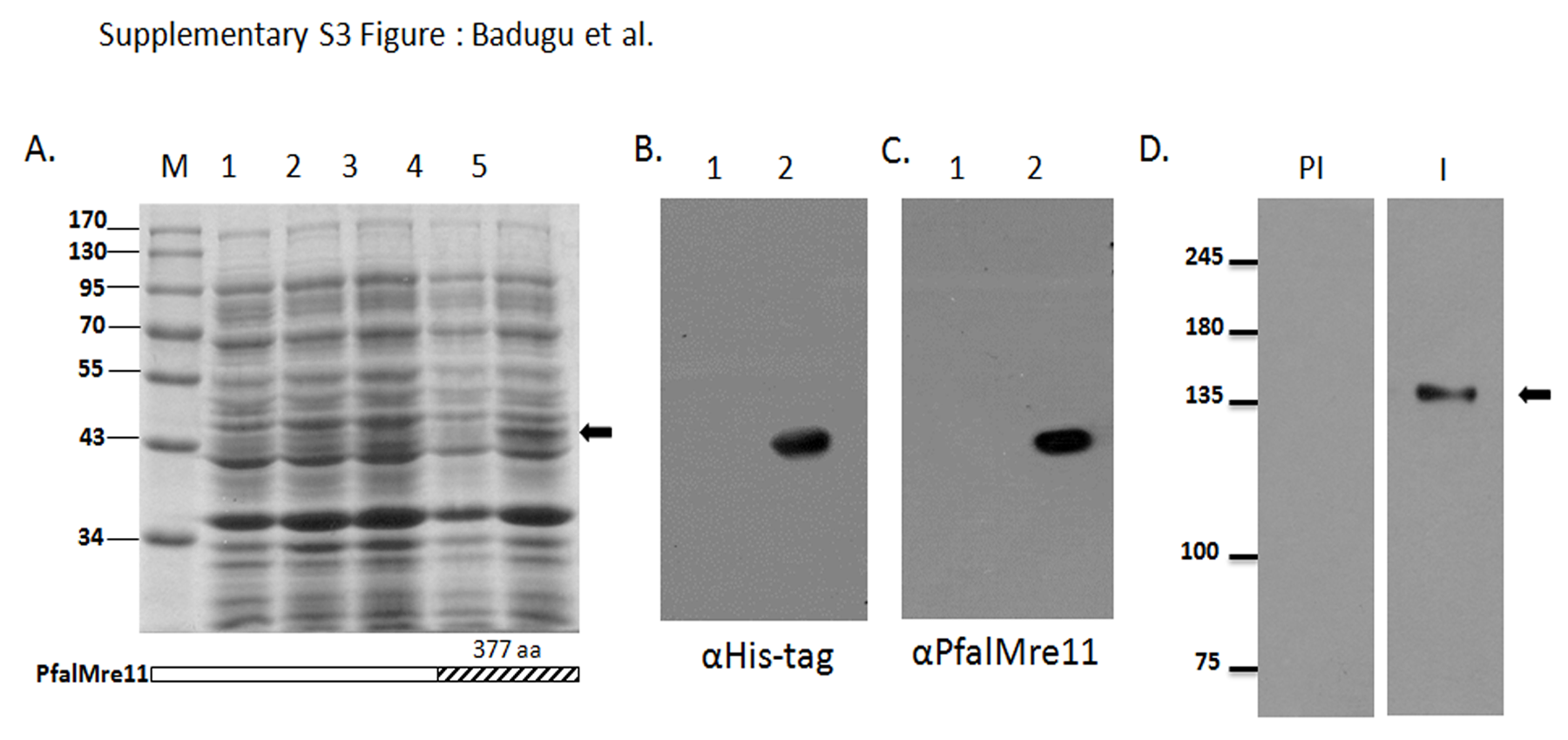

Supplement: S3 Fig — (A). A polypeptide corresponding to the last 377 amino-acids of PfalMre11 (as indicated by the hatched box) was expressed as an N-terminal His-tagged protein (marked by an arrow). Expression profile from cell lysates taken at 0 hour (lane 1); un-induced for 4 hour (lane 4) and induced with IPTG for 4 hours (lane 5) are shown. Lanes 2 and 3 represent protein profile from cells carrying empty vector and induced with IPTG for 4 hours or un-induced for 4 hours, respectively. Protein molecular weight markers (M) are indicated on the left side. (B). The expressed recombinant protein is recognized by anti-His antibody. Lane 1: empty vector; and lane 2: induced sample. (C). The anti-PfalMre11 antibody generated against the recombinant PfalMre11 protein (377 amino-acids at the C-terminal region) recognizes the recombinant protein from bacterial lysates. Lane 1: empty vector; and lane 2: induced sample. (D). The anti-PfalMre11 antibody detects a parasite protein of molecular weight 145 kDa (indicated by an arrow). PI: pre-immune sera; I: immune sera. (TIF) [file pone.0125358.s003.tif]

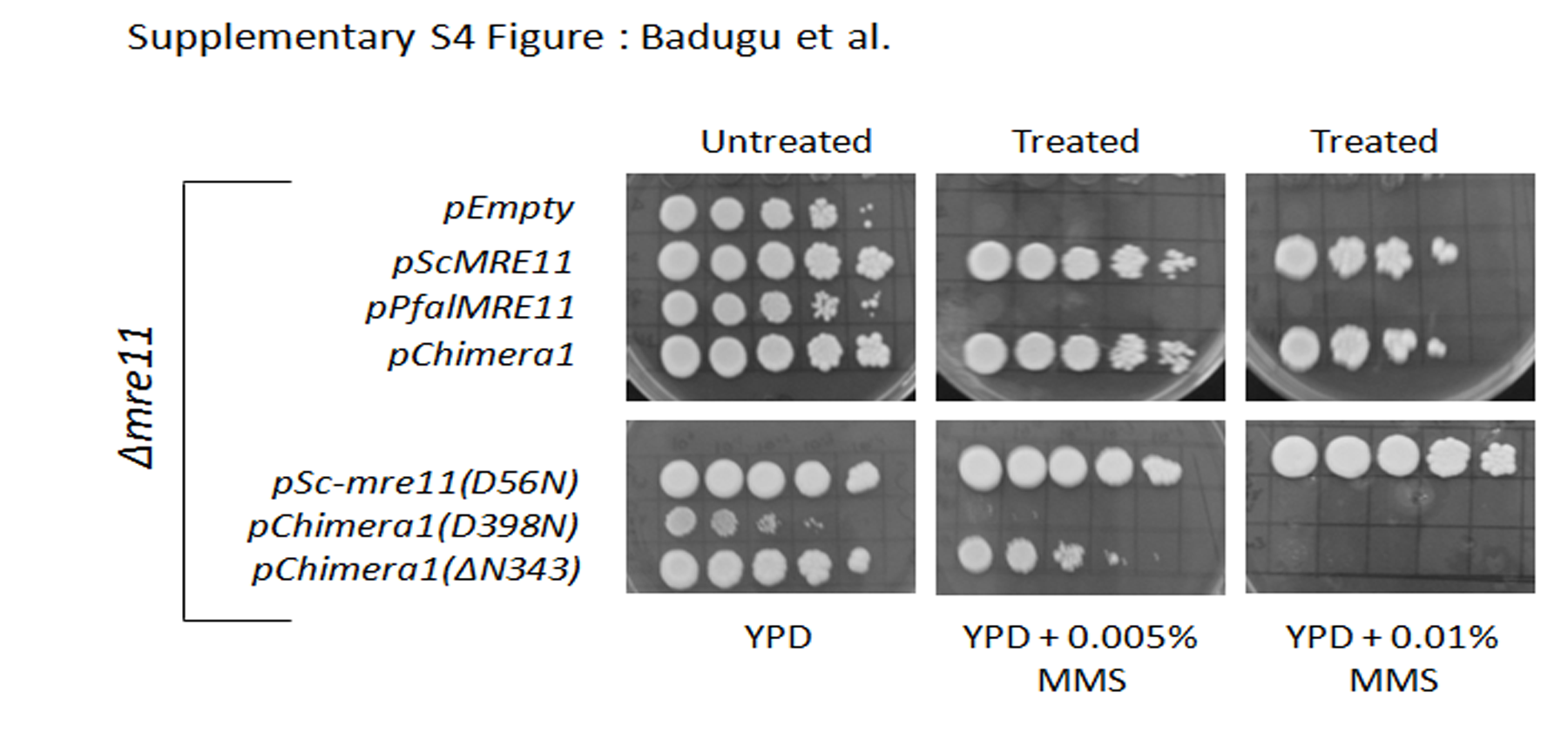

Supplement: S4 Fig — Spotting assays on YPD media without (untreated) or with (treated) 0.005% or 0.01% MMS supplementation are shown. The relevant genotypes are shown on the left. (TIF) [file pone.0125358.s004.tif]
