# Supplementary material for: Identification of Plasmodium falciparum DNA Repair Protein Mre11 with an Evolutionarily Conserved Nuclease Function
Source: PLoS One. 2015 May 4;10(5):e0125358. doi: 10.1371/journal.pone.0125358 (PMC4418825; doi:10.1371/journal.pone.0125358)
Supplement: S1 Table — (DOC) [file pone.0125358.s005.doc]

**Table S1. Primers used in this study.**

| **Primer** | **Sequence** | **Purpose** |
| --- | --- | --- |
| ***The following primers were used for cloning*** | | |
| OMKB23 | 5’-ATCCGGATCCATGAAAAGTACACAAAGTAATTT G-3’ | Forward primer to amplify full length *PfMRE11* |
| OMKB24 | 5’-ATCCGGATCCTTATTTTTTTCCTATGAGGTG-3’ | Reverse primer to amplify full length *PfMRE11* |
| OMKB84 | 5’-GGATCCATGGACTATCCTGATCCAGAC-3’ | Forward primer to amplify full length *ScMRE11* or *D56N* mutant |
| OMKB85 | 5’-CTGCAGCTATTTTCTTTTCTTAGCAAGGAGAC3’ | Reverse primer to amplify full length *ScMRE11* or *D56N* mutant |
| OMKB161 | 5’-GGATCCATGCATTTGAGGCCTCACGATAAAGAT G-3’ | Forward primer to amplify C- terminal domain of *ScMRE11* |
| OMKB100 | 5’-AAGCTTATAAGGTTTAGAAAAATGTTC-3’ | Reverse primer to amplify *PfMRE11* N- terminal domain |
| OMKB98 | 5’-AAGCTTCATTTGAGGCCTCACGATAAAGATG-3’ | Forward primer to amplify *ScMRE11* C- terminal domain |
| OMKB99 | 5’-GGATCCATGAGTAAAAATGATGCAAGTAC-3’ | Forward primer to amplify *Chimera 1 (∆N343)* |
| OMKB299 | 5’-ATTAAACAGTGGAAATTTATTTC-3’ | Overlapping primers for creating *D398N* mutation |
| OMKB298 | 5’-TTTATTTTTATGAAATAAATTTCC-3’ | Overlapping primers for creating *D398N* mutation |
| OMKB154 | 5’-AAGCTTATTTTTAATTGGATTGTGTAC-3’ | Reverse primer to amplify *ScMRE11* N- terminal domain |
| OMKB153 | 5’-AAGCTTTCTAAACCTTATTTAGAAGAAAG-3’ | Forward primer to amplify PfMRE11 C- terminal domain |
| OMKB89 | 5’-CTGCAGTTATTTTTTTCCTATGAGGTG-3’ | Reverse primer to amplify PfMRE11 C- terminal domain |
| OMKB188 | 5’- GGATCCATGAAAGATATAAAACTAGCTG-3’ | Forward primer to amplify *PfMRE11* C terminal domain |
| OMKB166 | 5’-GTCGACTTATTTTTTTCCTATGAGGTG-3’ | Reverse primer to amplify *PfMRE11* C terminal domain |
| OMKB262 | 5’-GGATCCATGTGGGTAGTACGATACCAG-3’ | Forward primer to amplify *ScXRS2* |
| OMKB263 | 5’-GGATCCTTATCCTTTTCTTCTTTTGAA-3’ | Reverse primer to amplify *ScXRS2* |
| OMKB164 | 5’-GGATCCATGAGCGCTATCTATAAATTATC-3’ | Forward primer to amplify *ScRAD50* |
| OMKB165 | 5’-GAATTCTCAATAAGTGACTCTGTTAATATC-3’ | Reverse primer to amplify *ScRAD50* |
| OMKB210 | 5’-GCGGCCGACGACAGCTTCCG-3’ | Forward primer to amplify *YKU80* |
| OMKB75 | 5’-CTACTCGACGAGATCGAGTAAGTC-3’ | Reverse primer to amplify *YKU80* |
| OMKB167 | 5’-GAATTCATGACTACGCTTGAAAAGATTG-3’ | Forward primer to amplify *PfRAD50* |
| OMKB326 | 5’-CTGCAGTCAAGTATTAACTCTTTCAATTTTTG-3’ | Reverse primer to amplify *PfRAD50* |
| ***The following primers were used for real-time RT-PCR*** | | |
| OSB139 | 5’-CAAATGTAACTTCTGATTTTACATC-3’ | Forward primer to amplify *PfMRE11* |
| OSB140 | 5’-AACATGTGAACAGCTACAATATG-3’ | Reverse primer to amplify *PfMRE11* |
| OSB94 | 5’-CTGTAACACATAATAGATCCGAC-3’ | Forward primer to amplify *PfARP* |
| OSB95 | 5’-TTAACCATCGTTATCATCATTATTTC-3’ | Reverse primer to amplify *PfARP* |
| OSB14 | 5’-TTAGAAACACTTGTGGTGAACG-3’ | Forward primer to amplify *ScACT1* |
| OSB16 | 5’-TGACCAAACTACTTACAACTCC-3’ | Reverse primer to amplify *ScACT1* |
| OMKB175 | 5’-AGATGAAGATGAAGGAGAC-3’ | Forward primer to amplify *PfMRE11* and *Chimera 2* |
| OMB77 | 5’-TTTTCTTTTCTTAGCAAGGAG-3’ | Forward primer to amplify *ScMRE11*, Chimera 1, *ScMRE11C*, *Chimera 1 (∆N343)* and *Chimera 1 (D398N)* |
| OSB50 | 5’-CAAACGAGTGCGAACTGCAAC-3’ | Reverse primer to amplify *ScMRE11*, Chimera 1, *ScMRE11C*, *Chimera 1 (∆N343)* and *Chimera 1 (D398N)* |
