# Supplementary material for: Identification of Plasmodium falciparum DNA Repair Protein Mre11 with an Evolutionarily Conserved Nuclease Function
Source: PLoS One. 2015 May 4;10(5):e0125358. doi: 10.1371/journal.pone.0125358 (PMC4418825; doi:10.1371/journal.pone.0125358)
Supplement: S2 Table — (DOC) [file pone.0125358.s006.doc]

**Table S2. Yeast strains used in this study.**

| ***Strain*** | ***Genotype*** | ***Reference*** |
| --- | --- | --- |
| W303α | *MATα leu2-3, 112 his3-11, 15 ade2-1, trp1, ura3-1* | [18] |
| MKB4 | *MATa leu2-3, 112 his3-11, 15 ade2-1, trp1, ura3-1, mre11D56N ::URA3, VIIL::ADE2 (pSD160)* | [18] |
| MKB7 | *MATa leu2-3, 112 his3-11, 15 ade2-1, trp1, ura3-1, mre11Δ::URA3, VIIL::ADE2 (pSD160), LEU2 (pSD158)* | [18] |
| BSB1 | *MATα leu2-3, 112 his3-11, 15 ade2-1, trp1, ura3-1, pTA* | This work |
| BSB2 | *MATa leu2-3, 112 his3-11, 15 ade2-1, trp1, ura3-1, mre11Δ::URA3, VIIL::ADE2 (pSD160), LEU2 (pSD158), pTA/ ScMRE11* | This work |
| BSB3 | *MATa leu2-3, 112 his3-11, 15 ade2-1, trp1, ura3-1, mre11Δ::URA3, VIIL::ADE2 (pSD160), LEU2 (pSD158), pTA/ PfalMRE11* | This work |
| BSB4 | *MATa leu2-3, 112 his3-11, 15 ade2-1, trp1, ura3-1, mre11Δ::URA3, VIIL::ADE2 (pSD160), LEU2 (pSD158), pTA/ Chimera 1* | This work |
| BSB5 | *MATa leu2-3, 112 his3-11, 15 ade2-1, trp1, ura3-1, mre11Δ::URA3, VIIL::ADE2 (pSD160), LEU2 (pSD158), pTA/ Chimera 2* | This work |
| BSB6 | *MATa leu2-3, 112 his3-11, 15 ade2-1, trp1, ura3-1, mre11Δ::URA3, VIIL::ADE2 (pSD160), LEU2 (pSD158), pTA/ ScMRE11C* | This work |
| BSB7 | *MATa leu2-3, 112 his3-11, 15 ade2-1, trp1, ura3-1, mre11Δ::URA3, VIIL::ADE2 (pSD160), LEU2 (pSD158), pTA/ Chimera 1 (∆N343)* | This work |
| BSB8 | *MATa leu2-3, 112 his3-11, 15 ade2-1, trp1, ura3-1, mre11Δ::URA3, VIIL::ADE2 (pSD160), LEU2 (pSD158), pTA* | This work |
| PVY1 | *MATa leu2-3, 112 his3-11, 15 ade2-1, trp1, ura3-1, mre11Δ::URA3, VIIL::ADE2 (pSD160), LEU2 (pSD158), pTA/ Chimera 1 (D398N)* | This work |
| PVY2 | *MATa leu2-3, 112 his3-11, 15 ade2-1, trp1, ura3-1, mre11Δ::URA3, VIIL::ADE2 (pSD160), LEU2 (pSD158), pTA/mre11(D56N)* | This work |
| PJ69-4A | *MATa trpl-901 leu2-3,112 ura3-52 his3-200 ga14∆ ga180∆ LYS2 :: GALl-HIS3 GAL2-ADE2, met2::GAL7-lacZ* | [34] |
| BSB14 | *MATa trpl-901 leu2-3,112 ura3-52 his3-200 ga14∆ ga180∆ LYS2 :: GALl-HIS3 GAL2-ADE2, met2::GAL7-lacZ pGBDUC1* | This work |
| BSB15 | *MATa trpl-901 leu2-3,112 ura3-52 his3-200 ga14∆ ga180∆ LYS2 :: GALl-HIS3 GAL2-ADE2, met2::GAL7-lacZ pGBDUC1 / PfalMRE11* | This work |
| BSB18 | *MATa trpl-901 leu2-3,112 ura3-52 his3-200 ga14∆ ga180∆ LYS2 :: GALl-HIS3 GAL2-ADE2, met2::GAL7-lacZ pGBDUC1, pGADC1* | This work |
| BSB19 | *MATa trpl-901 leu2-3,112 ura3-52 his3-200 ga14∆ ga180∆ LYS2 :: GALl-HIS3 GAL2-ADE2, met2::GAL7-lacZ pGBDUC1, pGADC1/ PfalMRE11* | This work |
| BSB20 | *MATa trpl-901 leu2-3,112 ura3-52 his3-200 ga14∆ ga180∆ LYS2 :: GALl-HIS3 GAL2-ADE2, met2::GAL7-lacZ pGBDUC1/ PfalMRE11, pGADC1* | This work |
| BSB21 | *MATa trpl-901 leu2-3,112 ura3-52 his3-200 ga14∆ ga180∆ LYS2 :: GALl-HIS3 GAL2-ADE2, met2::GAL7-lacZ pGBDUC1/ PfalMRE11, pGADC1/ PfalMRE11* | This work |
| BSB22 | *MATa trpl-901 leu2-3,112 ura3-52 his3-200 ga14∆ ga180∆ LYS2 :: GALl-HIS3 GAL2-ADE2, met2::GAL7-lacZ pGBDUC1/ ScXRS2* | This work |
| BSB23 | *MATa trpl-901 leu2-3,112 ura3-52 his3-200 ga14∆ ga180∆ LYS2 :: GALl-HIS3 GAL2-ADE2, met2::GAL7-lacZ pGBDUC1/ ScXRS2, pGADC1* | This work |
| BSB24 | *MATa trpl-901 leu2-3,112 ura3-52 his3-200 ga14∆ ga180∆ LYS2 :: GALl-HIS3 GAL2-ADE2, met2::GAL7-lacZ pGBDUC1/ ScXRS2, pGADC1/ ScMRE11* | This work |
| BSB25 | *MATa trpl-901 leu2-3,112 ura3-52 his3-200 ga14∆ ga180∆ LYS2 :: GALl-HIS3 GAL2-ADE2, met2::GAL7-lacZ pGBDUC1/ ScXRS2, pGADC1/ PfalMRE11* | This work |
| BSB26 | *MATa trpl-901 leu2-3,112 ura3-52 his3-200 ga14∆ ga180∆ LYS2 :: GALl-HIS3 GAL2-ADE2, met2::GAL7-lacZ pGBDUC1/ ScXRS2, pGADC1/ chimera 1* | This work |
| BSB28 | *MATa trpl-901 leu2-3,112 ura3-52 his3-200 ga14∆ ga180∆ LYS2 :: GALl-HIS3 GAL2-ADE2, met2::GAL7-lacZ pGBDUC1/PfalRAD50 pGADC1* | This work |
| BSB30 | *MATa trpl-901 leu2-3,112 ura3-52 his3-200 ga14∆ ga180∆ LYS2 :: GALl-HIS3 GAL2-ADE2, met2::GAL7-lacZ pGBDUC1/PfalRAD50 pGADC1/PfalMRE11* | This work |
| BSB32 | *MATa trpl-901 leu2-3,112 ura3-52 his3-200 ga14∆ ga180∆ LYS2 :: GALl-HIS3 GAL2-ADE2, met2::GAL7-lacZ pGBDUC1/PfalMRE11 pGADC1/ScMRE11* | This work |
| BSB33 | *MATa trpl-901 leu2-3,112 ura3-52 his3-200 ga14∆ ga180∆ LYS2 :: GALl-HIS3 GAL2-ADE2, met2::GAL7-lacZ pGBDUC1/ScMRE11 pGADC1/ScMRE11* | This work |
| SAN1 | *MATa trpl-901 leu2-3,112 ura3-52 his3-200 ga14∆ ga180∆ LYS2 :: GALl-HIS3 GAL2-ADE2, met2::GAL7-lacZ pGBDUC1/ScRAD50* | This work |
| SAN2 | *MATa trpl-901 leu2-3,112 ura3-52 his3-200 ga14∆ ga180∆ LYS2 :: GALl-HIS3 GAL2-ADE2, met2::GAL7-lacZ pGBDUC1/ ScRAD50, pGADC1* | This work |
| SAN3 | *MATa trpl-901 leu2-3,112 ura3-52 his3-200 ga14∆ ga180∆ LYS2 :: GALl-HIS3 GAL2-ADE2, met2::GAL7-lacZ pGBDUC1/ ScRAD50, pGADC1/ PfalMRE11* | This work |
| SAN4 | *MATa trpl-901 leu2-3,112 ura3-52 his3-200 ga14∆ ga180∆ LYS2 :: GALl-HIS3 GAL2-ADE2, met2::GAL7-lacZ pGBDUC1/ ScRAD50, pGADC1/ ScMRE11* | This work |
| SAN5 | *MATa trpl-901 leu2-3,112 ura3-52 his3-200 ga14∆ ga180∆ LYS2 :: GALl-HIS3 GAL2-ADE2, met2::GAL7-lacZ pGBDUC1/ ScRAD50; pGADC1/ chimera 1* | This work |
| SAN7 | *MATa trpl-901 leu2-3,112 ura3-52 his3-200 ga14∆ ga180∆ LYS2 :: GALl-HIS3 GAL2-ADE2, met2::GAL7-lacZ pGBDUC1/YKU80 pGADC1* | This work |
| SAN8 | *MATa trpl-901 leu2-3,112 ura3-52 his3-200 ga14∆ ga180∆ LYS2 :: GALl-HIS3 GAL2-ADE2, met2::GAL7-lacZ pGBDUC1/YKU80 pGADC1/ScMRE11* | This work |
| SAN9 | *MATa trpl-901 leu2-3,112 ura3-52 his3-200 ga14∆ ga180∆ LYS2 :: GALl-HIS3 GAL2-ADE2, met2::GAL7-lacZ pGBDUC1/YKU80 pGADC1/PfalMRE11* | This work |
| SAN10 | *MATa trpl-901 leu2-3,112 ura3-52 his3-200 ga14∆ ga180∆ LYS2 :: GALl-HIS3 GAL2-ADE2, met2::GAL7-lacZ pGBDUC1/YKU80 pGADC1/ chimera 1* | This work |
